# Metastasis Initiation Precedes Detection of Primary Cancer—Analysis of Metastasis Growth *in vivo* in a Colorectal Cancer Test Case

**DOI:** 10.3389/fphys.2020.533101

**Published:** 2020-12-17

**Authors:** Gili Hochman, Einat Shacham-Shmueli, Stephen P. Raskin, Sara Rosenbaum, Svetlana Bunimovich-Mendrazitsky

**Affiliations:** ^1^Department of Mathematics, Ariel University, Ariel, Israel; ^2^Sheba Medical Center, Tel Hashome, Israel

**Keywords:** lung metastases, mathematical growth models, primary tumor resection, exponential growth, logistic growth, liver metastasectomy, colorectal cancer, clinical metastasis growth data

## Abstract

Most cases of deaths from colorectal cancer (CRC) result from metastases, which are often still undetectable at disease detection time. Even so, in many cases, shedding is assumed to have taken place before that time. The dynamics of metastasis formation and growth are not well-established. This work aims to explore CRC lung metastasis growth rate and dynamics. We analyzed a test case of a metastatic CRC patient with four lung metastases, with data of four serial computed tomography (CT) scans measuring metastasis sizes while untreated. We fitted three mathematical growth models—exponential, logistic, and Gompertzian—to the CT measurements. For each metastasis, a best-fitted model was determined, tumor doubling time (TDT) was assessed, and metastasis inception time was extrapolated. Three of the metastases showed exponential growth, while the fourth showed logistic restraint of the growth. TDT was around 93 days. Predicted metastasis inception time was at least 4–5 years before the primary tumor diagnosis date, though they did not reach detectable sizes until at least 1 year after primary tumor resection. Our results support the exponential growth approximation for most of the metastases, at least for the clinically observed time period. Our analysis shows that metastases can be initiated before the primary tumor is detectable and implies that surgeries accelerate metastasis growth.

## Introduction

Colorectal cancer (CRC) is one of the most common causes of cancer-related deaths worldwide, and the primary cause for CRC patient death is the development of metastatic disease (Van Cutsem et al., 2014; Vatandoust et al., 2015). Statistical data are available on patterns of colorectal metastasis sites (Riihimäki et al., 2016; Stewart et al., 2018), but the dynamics of metastasis formation and growth are not well-established. It is assumed that a significant part of metastases is seeded at a very early stage, before primary tumor detection (Fisher et al., 1977; Fisher, 1980; Siegel et al., 2017). Surgery is now the main curative treatment in both local and metastatic diseases (Stein and Schlag, 2007).

The most common site of CRC metastases is the liver, and the next is the lungs. Liver resection is now the standard of care for patients with resectable hepatic metastases (Stewart et al., 2018). However, there is evidence that stress response aroused by surgery may accelerate metastasis growth (Behrenbruch et al., 2018; Zheng et al., 2018). The extrahepatic disease is considered a risk factor in terms of survival after hepatic metastasectomy (Stewart et al., 2018). Specifically, the presence of limited preoperative small pulmonary nodules in the lungs was claimed to be associated with shorter progression-free survival (PFS) after hepatic metastasectomy (Maithel et al., 2010). Data on the effects of such a surgery on the growth of remaining metastases is not available and cannot be deduced retrospectively. Mathematical models, providing reliable representation of the metastasis growth patterns, may shed light on the metastatic growth process, and help in optimizing treatments for the prevention of metastasis growth.

Mathematical growth models are used as simplified approximations to dynamics of the actual biological process. Such models were extensively studied for primary tumors (Brú et al., 2003; Kozusko and Bajzer, 2003), but much less for metastasis growth dynamics. When modeled, exponential growth is often assumed, at least for the first period of growth (Haeno et al., 2012; Benzekry et al., 2014; Hanin and Bunimovich-Mendrazitsky, 2014; Hanin et al., 2016), although logistic or Gompertzian models—which have the feature of upper limitation on growth—are biologically more plausible. Comparison of different growth laws had been done by modeling *in vivo* data of metastatic cancer in several works, starting with Iwata's model (Iwata et al., 2000). When this model was applied on hepatocellular carcinoma patient data, the Gompertzian growth showed the best fit for the dynamics and size distribution of multiple liver metastases. Other works that followed are mostly based on animal models, for which data of untreated metastases is easier to obtain than for humans. See Hartung et al. (2014), Baratchart et al. (2015), Benzekry et al. (2016), and lately, Vaghi et al. (2020), who suggested that the Gompertzian growth model is the most appropriate model to be used for predictions of the metastatic growth process.

However, such predictions are hard to prove in humans, since clinical data on untreated metastasis growth is rare. Added to the diversity between different patients and metastases, it increases the difficulty in finding reliable growth patterns to be used as predictors. Specifically, for pulmonary metastases, the available clinical data implies that in most cases, exponential growth is a good enough approximation for the time period of observation (Collins et al., 1956; Sabra et al., 2017). Yet, different types of pulmonary metastases may vary in their growth pattern, in the natural history of the disease, and also in the possible different effects of surgery on the growth of the remaining metastases. Hence, the analysis of longitudinal clinical data of specific metastases dynamics is essential in order to characterize metastasis growth and pave the way to individualized prognosis and therapy.

Lately, we have published an analysis of data from a rare test case of a metastatic CRC patient, with untreated growth of 10 lung metastases repeatedly measured over 3 years (Hochman et al., 2019). We have shown that exponential growth can be approximated to all metastases and that metastases were initiated at least 8–11 years before the primary disease detection. Here, we present another unique test case of a colon cancer patient with measured growth of untreated lung metastases. These metastases were first detected 2.6 years after primary tumor resection, and 1.7 years after a liver metastasis was resected in a second operation. This case is different from the former (Hochman et al., 2019) in the location of the primary tumor—sigmoid colon in this case, and rectal in the former case, and also in the fact that here there were two metastatic locations (liver and lungs), and two operations were conducted. These distinctions imply a different type of lung metastases, with a possibly different route of metastatic spread, which may induce a different course of the natural history of metastases. We analyze the current case in the same way as in the former case, examining the validity of exponential, logistic, and Gompertzian approximations, and estimate the natural history (i.e., time of onset) of metastases. We address the question of whether former conclusions are also valid for this case.

In addition, the primary tumor, in this case, was detected and removed relatively early, at the size of 0.68 cm^3^, when the liver and lung metastases were still undetectable. This is compared to the former case, where at the time of disease detection there was a 6-cm^3^ tumor in the rectum, a colonic polyp, and at least eight lung metastases were already detectable. Here, we re-examine the prediction of early metastasis onset time, not only for metastases observed at first detection of the primary (as in Hochman et al., 2019) but also for metastases that were occult at the time of disease detection and observed only 2 years afterward. In this case of late-detected metastases, we also faced a more difficult task as we wanted to study the effects of the two surgeries on metastasis growth.

## Methods

### Data

**Figure 1:**
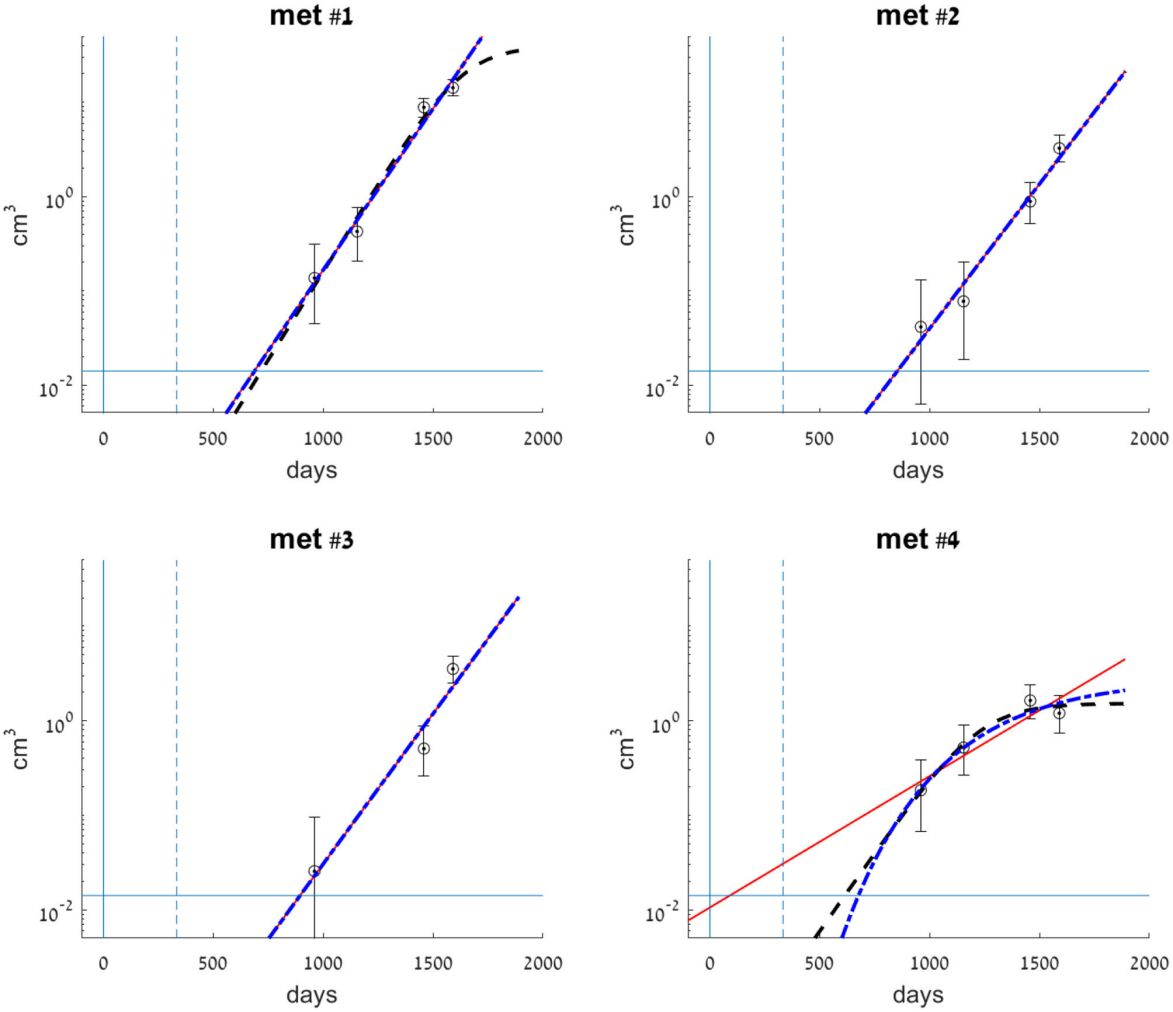
Clinical data measurements (circles) for each of the observed metastases, compared with exponential (smooth red line), logistic (dashed black line), and Gompertzian (dashed-dotted blue line) growth laws fitted to the data. Metastases volumes are presented on a logarithmic scale. Model parameters for each of the metastases were fitted to its observed volumes (see Supplementary Table 1). Error bars for each of the clinical data measurements represent errors in volume, where the measurement error is ±2 mm in each dimension of the lesion. Vertical lines at *t* = 0 (smooth) and *t* = 333 (dashed) mark the days of primary colon tumor resection and liver metastasis resection, respectively. The horizontal line at 0.014 cm^3^ marks the detection limit of the CT scan.

A 59-year-old patient was diagnosed with sigmoid colon cancer and underwent resection, revealing stage T2N1M0. The measured volume of the primary tumor at surgery was 0.68 cm^3^. Adjuvant chemotherapy was given (5FU for 6 months and oxaliplatin, which was stopped after one cycle because of allergic reaction). One year after primary tumor removal, a metastasis was discovered in the liver and was resected. CT scans at the time of the first diagnosis and at the time of liver metastasectomy did not detect any metastases in the lungs. After liver metastasectomy (1.7 years), chest CT showed four metastases. Three additional scans were conducted in the following 2 years. The measured volumes of metastases at these time points are reported in Supplementary Table 1 and visualized in Figure 1. Metastases were peripheral, with no large vessels observed near them, which negates vascularization effects on metastasis growth rate. During this period, systemic treatment (chemotherapy, targeted treatment) was not administered due to patient preference.

### Modeling

Based on the measurement data obtained, we wanted to fit a growth model (exponential, logistic, or Gompertzian) for each of the metastases and assess growth rate parameter values using the same methods described in Hochman et al. (2019).

Exponential growth was modeled by:

(1) $$\begin{array}{l} \text{Ψ}\left(\text{t}\right)={\text{N}}_{0}^{\exp}e^{\lambda t}, \end{array}$$

where Ψ(t) is the metastasis volume at time t, counted from the day of primary tumor resection, ${\text{N}}_{0}^{\exp}$ is the size of metastasis at *t* = 0, and λ is the growth rate parameter.

Logistic growth was modeled by:

(2) $$\begin{array}{l} \Theta \left(t\right)=\;\frac{K^{logistic}}{1+\left(\frac{K^{logistic}}{{\text{N}}_{0}^{logistic}}-1\right)e^{-rt}}, \end{array}$$

where Θ(t) is metastasis volume at time t, $N_{0}^{logistic}$ is the size of metastasis at *t*=0, *K*^*logistic*^ is the upper limit of tumor size (carrying capacity), and r is a rate parameter.

Gompertzian growth was modeled by:

(3) $$\begin{array}{l} \Phi \left(t\right)=\;K^{gomp}e^{\ln\left(\frac{{\text{N}}_{0}^{gomp}}{K^{gomp}}\right)e^{-\beta t}}, \end{array}$$

where Φ(*t*) is metastasis volume at time t, ${\text{N}}_{0}^{gomp}$ is the size of metastasis at *t*=0, *K*^*gomp*^ is the limiting tumor size, and β is a rate parameter.

Tumor doubling time (TDT) can be calculated, in the case of exponential growth, from the growth rate parameter (λ in Equation 1), using the equation:

(4) $$\begin{array}{l} TDT=ln\left(2\right)/\lambda . \end{array}$$

In case of logistic growth, when Θ(*t*) ≪ *K*^*logistic*^, an approximation of Equation 2 gives *TDT* the same as in Equation 4, with *r* instead of λ.

The direct fit of the data was carried out for each of the metastases separately, to optimize the parameter values by numerical minimization of the sum of squared errors (SSE) for model predictions compared to the log-volume of measured tumor sizes:

(5) $$\begin{array}{l} SSE=\sum_{i=1}^{n}{\left(ln\left(f\left(t_{i},p\right)\right)-ln\left(Y_{i}\right)\;\right)}^{2},\; \end{array}$$

where *n* is the total number of available measurements, *Y*_*i*_ is the observed metastasis volume at time *t*_*i*_, and *f*(*t*_*i*_, *p*) is the predicted metastasis volume at the same time, as calculated by each of the model equations (Equations 1–3), depending on the estimated parameters vector *p*, which includes the two or three parameters of the relevant model equation.

The search was limited to biologically feasible parameter values: ${\text{N}}_{0}^{\exp}$ ≥0, λ≥0 In Equation 1, ${\text{N}}_{0}^{logistic}$ ≥0, *r*≥0, *K*^*logistic*^ ≥1 *cell volume* in Equation 2, and ${\text{N}}_{0}^{gomp}$ ≥0, β ≥0, *K*^*gomp*^≥10^−9^cm^3^ in Equation 3. Note, that for all three models, values of ${0<\text{N}}_{0}<10^{-9}{\;cm}^{3}\;=\;1cellvolume$ mean that the time of inception of metastasis (defined as time of appearance of the first malignant cell) is after the time of tumor resection, defined at *t* = 0.

We also assume a minimal biologically plausible value for metastasis doubling time. According to the reported statistical data, the range of TDT values starts from 28.2 days (Tomimaru et al., 2018) or even 22 days (Chojniak and Younes, 2003), as measured in groups of 65 and 21 patients with CRC pulmonary metastases, respectively. Therefore, we limited the selection of parameter values to obtain a minimum TDT value of 25 days, from the time of the onset of metastasis until the time when the threshold volume for detection by CT scan was reached. This threshold is approximated as 0.014 cm^3^, which is the volume of a spherical lesion with a 3-mm diameter (Bankier et al., 2017). The procedure was performed using the Matlab functions *lsqnonlin, nlinfit*, and *fmincon*.

Subsequently, the fitted models were used to estimate the time of onset of metastasis. For this purpose, the fitted curve with estimated parameters for each metastasis *k* was extrapolated backward to determine the time of onset of metastasis (*T*_*k*_), defined as the time of appearance of the first malignant cell. In the same way, we assessed the earliest possible detection time (*D*_*k*_), defined as the time of metastasis size reaching the threshold enabling detection by CT scan, defined above as 0.014 cm^3^.

### Error Estimation

The maximal experimental error in measuring the metastatic volumes was ±2 mm in each dimension of the lesion, which is assumed to be spherical. For each reported data point, we calculated the measured diameter of a sphere, and the measurement error in volume (reflected in the error bars in Figure 1) was estimated according to this measured diameter ±2 mm.

To assess the reliability of the fitted models within the measurement errors, a sensitivity analysis was conducted, by simulating 1,000 random samples of artificial data, uniformly distributed within these error bars. For each of these samples, we have performed the model fit and obtained parameter values. Then, we have analyzed the distribution of the resulting fitted parameter values and of the estimated times for metastasis formation (*T*_*k*_) and metastasis earliest detection time (*D*_*k*_), which are directly defined by the fitted parameters. The mean, median, relative standard error, and interdecile range (i.e., difference between the first and the ninth deciles, 10 and 90%) of the fitted parameter values were calculated.

## Results

### Fitting and Comparing Growth Models

We have fitted each of the growth models examined to each of the four metastases. Values for the parameters of each of the three models were fitted to the dataset of all three or four available measurements in time. The parameters' optimal values are presented in Table 1. Fitted curves are presented, along with clinical measurements in Figure 1. The SSE score of the goodness of fit is also detailed in Table 1.

**Table 1:** Values of estimated optimal parameters for each of the analyzed metastases, of the three fitted models, along with the value of SSE (Equation 5).

	**Exponential**			**Logistic**				**Gompertz**			
	**${\text{N}}_{0}^{\text{e}x\text{p}}\left[{\text{cm}}^{3}\right]$**	**λ[years^−1^]**	**SSE**	**${\text{N}}_{0}^{\text{l}ogisti\text{c}}\left[{\text{cm}}^{3}\right]$**	**K^logistic^[cm^3^]**	**r[years^−1^]**	**SSE**	**${\text{N}}_{0}^{\text{g}om\text{p}}\left[{\text{cm}}^{3}\right]$**	**K^gomp^[cm^3^]**	**β[years^−1^]**	**SSE**
met #1	6.35E−05	2.87	0.26	2.86E−05	39.23	3.15	0.22	5.91E−05	2.84E+296	4.21E−03	0.26
met #2	3.42E−05	2.58	0.36	3.42E−05	7.10E+06	2.58	0.36	3.24E−05	6.11E+307	3.63E−03	0.36
met #3	2.03E−05	2.67	0.49	2.03E−05	2.35E+07	2.67	0.49	1.92E−05	6.30E+307	3.76E−03	0.50
met #4	0.0106	1.17	0.36	1.29E−04	1.52	2.80	0.09	9.23E−12	2.81	0.87	0.14

*In case of small or no difference between the Gompertzian or Logistic model and the exponential one, we chose the exponential model, which is simpler and more reliable. Note, that for metastasis #3, there were only three data points available; hence, models with three parameters (i.e., Gompertzian and logistic) should reproduce three data points exactly. However, since we have limited the numerical search to feasible values (see section Materials and Methods), the fit of Gompertzian and logistic models converged to exponential growth*.

*Colored lines represent the chosen models, for which the sum of squared error (SSE) value was smallest*.

Metastases #1–3 were constantly growing over the entire time period examined. In general, the exponential growth model provided a good fit for these metastases (see Figure 1). Logistic and Gompertzian models were, in most cases, redundant; they converge with extremely high values of the parameter *K* (see Table 1), i.e., they essentially degenerate into an exponential model. For metastasis #1, the logistic model showed a slightly better fit than the exponential model (with lower SSE value, Table 1). However, since the exponential model is simpler, i.e., with one less parameter, and since the difference between the two models' predictions in the time period of interest (to the date of the last measure) was small, we considered also this metastasis as exponentially growing. Furthermore, sensitivity analysis has shown that logistic model parameter values are more sensitive to changes in the measured sizes within the measurement errors (see Supplementary Table 3, rows 5–9 compared to rows 1–4 and Supplementary Figures 1–11). Therefore, the exponential model is more reliable in this case.

For metastasis #4, the last measure showed growth had stopped; hence, the exponential model demonstrated poor accuracy. Gompertzian model is not reliable in this case, as its parameters optimal values are very sensitive to changes in data (see sensitivity analysis results, Supplementary Table 3, last five rows, and Supplementary Figures 12–17). The logistic model yielded the best fit to actual growth measurements (SSE value, Table 1). Note that the data shows a slight decrease in volume; however, this decrease is within the measurement error range. Therefore, the fitted logistic model shows that the metastasis' volume has reached its capacity, and the fitted value of *K*^*logistic*^ (Table 1) is close to the last two measured values (and within the measurement error range, as shown in Figure 1).

In conclusion, for metastases #1–3, the exponential growth model is the preferable one, while for metastasis #4, the logistic model showed the best fit.

### Metastasis Growth Rate

For the exponentially growing metastases (#1–3), the values of the exponent of the growth rate λ are all in the same order of magnitude, averaged 2.71 years^−1^, with a standard deviation of 0.15 year^−1^. This value corresponds to a tumor doubling time of 93 days (Equation 4). For metastasis #4, TDT can be approximated for the first period, when growth is close to exponential. In this case, the logistic growth rate is represented by the parameter r in Equation 2. Its fitted value was 2.80 years^−1^, corresponding to TDT = 90 days, which is close to the exponential growth rate of metastases #1–3.

### Assessing Metastasis Natural History

**Figure 2:**
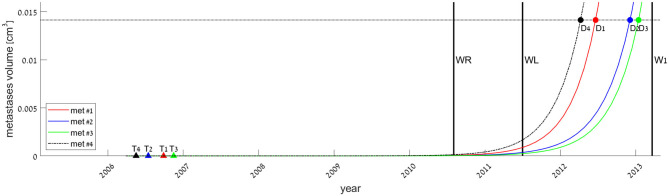
Estimated times of onset of metastases, defined as the time of appearance of the first malignant cell (filled triangles, marked *T*_*k*_ for each metastasis #*k*), and estimated times of metastases' earliest possible detection time, defined as the time of metastasis size reaching the threshold enabling detection by CT scan (filled circles, *D*_*k*_). *T*_*k*_ and *D*_*k*_ values were extrapolated from the fitted models, exponential (smooth lines, for metastases #1–3) or logistic (dashed-dotted, for metastasis #4). Vertical lines represent the dates of primary tumor resection (WR), liver metastasis resection (WL), and first measure of lung metastases (W1). The horizontal line at 0.014 cm^3^ represents the detection limit of the CT scan.

If we assume each metastasis has followed the same growth law since its inception, then the metastasis onset time (i.e., time of emergence of the first malignant clonogenic cell), *T*_*k*_, can be estimated for each metastasis #k. Backward extrapolation of the fitted growth curves can be used to find the time when metastasis volume is one cell, according to the model. The earliest possible detection time (i.e., time of metastasis size reaching to the threshold enabling detection by CT scan), *D*_*k*_, can be evaluated in the same way, extrapolating to the time when tumor size according to the model is 0.014 cm^3^. This extrapolation, according to the best-fitted growth curve—logistic for metastasis #4 and exponential for the others—is presented in Figure 2. Calculated values for *T*_*k*_ and *D*_*k*_ for every metastasis, by each of the three fitted models, are presented in Supplementary Table 2. The results show that all metastases were formed around 4 years before the primary tumor was detected. Yet, the earliest possible time when a metastasis could be detected was only after the second (liver metastasis) resection, marked as *D*_1_*-D*_4_ in Figure 2.

Note that the calculated values for *T*_4_ and *D*_4_ based on the logistic model are more sensitive to the measurement error than those based on exponential fit (see Supplementary Table 3, rows 6–7 from the end). Hence, the latter conclusion should not be taken as certain for metastasis #4.

## Discussion

Understanding metastasis dynamics and growth is essential for improving cancer therapy, especially toward individualization of treatment. Retrospective statistical data can recognize patterns of metastasis growth in different subgroups of patients but cannot decipher the reasons for the difference between subgroups. Analysis of specific cases, particularly utilizing clinical dynamical data of metastasis growth, is necessary to gain a deeper understanding of the metastatic process, and eventually provide reliable individual prognosis and treatment plans. In this work, we used unique data of a metastatic CRC patient to explore the dynamics of untreated lung metastasis growth. We concluded the natural history of the disease and how it is affected by factors like surgical intervention.

**Figure 3:**
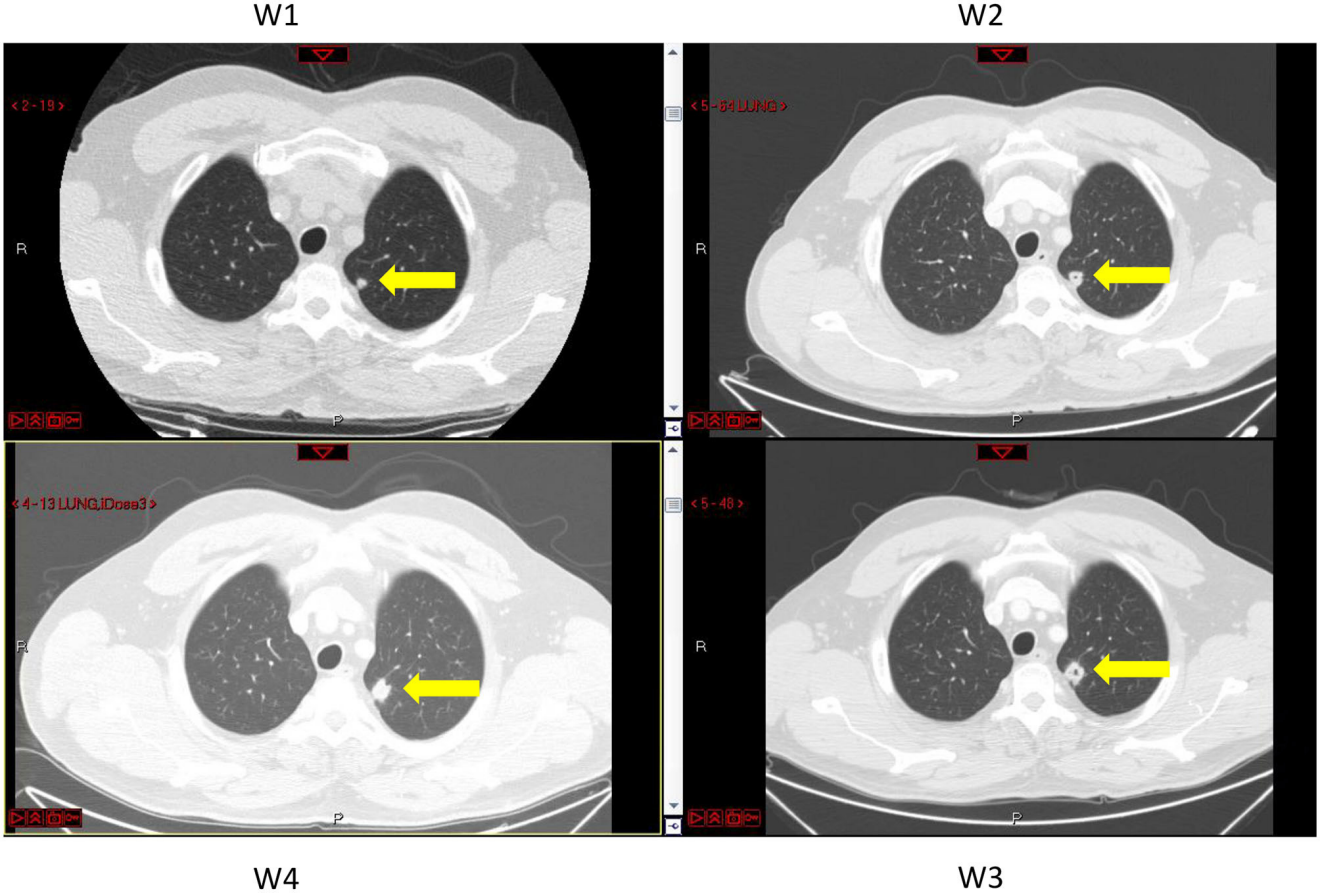
CT images of the lungs taken at different times, marked W1 to W4, at which metastasis volumes were measured (see Figure 1 and Supplementary Table 1). Metastasis #4 is marked by yellow arrows. A pore in the center of the lesion can be seen at times W2 and W3.

In the test case examined here, three lung metastases (metastases #1–3) constantly grew, and for them, *exponential growth* was found to be a good approximation. The estimated exponential growth rates of all metastases were quite similar, implying that variability between metastasis growth rates can be neglected. This result agrees with the former analyzed case (Hochman et al., 2019). For the fourth metastasis, it seems that growth has stopped during the time period in which measures were taken (Figure 1), showing that the metastasis growth ability has reached a certain limit. This blockage of the increase was observed in parallel with cavitation formation in the lesion, as observed on the CT scans (Figure 3). In this case, the cavity volume is included in the reported measured volume. However, it forms a negligible portion of the lesion volume; hence, the halt in growth is not a direct effect of the cavitation. Yet, cavitary lesions may behave differently, as they are composed of heterogeneous tissue. Here, any unknown process that causes the observed deceleration of growth is implicitly modeled as a logistic decay of the metastasis growth rate.

In general, results imply that the metastatic growth is logistically bounded, although in most cases, exponential growth can be approximated for the time period of measures. This is in line with the exponential growth pattern that had been observed for pulmonary metastases from CRC (Collins et al., 1956) and thyroid cancer (Sabra et al., 2017).

*Metastasis average growth rate*, which is 2.71 years^−1^ for the exponentially growing metastases here, corresponding to TDT of 93 days, seems higher than the previously reported rate of 1.48 years^−1^ in Hochman et al. (2019) (TDT of 171 days). Heterogeneity of tumor aggressiveness is common among different patients. However, here we can surmise that the reason for this difference is related to the effect of the hepatic metastasectomy. In this case the patient underwent two surgeries, first to remove the primary tumor and later to remove the liver metastasis, in contrast to the formerly published case in which only one operation (for primary tumor resection) was conducted.

There is evidence that tumor resection has implications that accelerate the metastasis growth, both because of the stress response caused by surgery (Maida et al., 2009; Tohme et al., 2017; Behrenbruch et al., 2018; Zheng et al., 2018) and due to the removal of the inhibiting effect that the resected tumor had induced on metastases (Retsky et al., 2010; Benzekry et al., 2017; Hanin and Rose, 2018). From our data, we cannot determine what was the metastasis growth rate before the surgeries. However, we hypothesize that in the secondary (lung) metastases, during their growth, the patient undergoes two surgeries, growth rate increases even more than after a single surgery. Besides, the primary tumor was in the sigmoid colon; therefore, it is likely that metastatic spread was via the portal circulation to the liver, and not directly to the lungs (Riihimäki et al., 2016). We can hypothesize that metastases that have developed in a later stage, as a “metastasis of metastasis,” might represent a more aggressive phenotype.

The aggressiveness of metastases can be caused by other unknown variables. Hence, a general conclusion from a comparison between cases is limited. With that being said, this case can be compared to statistical data available in the literature for the TDT of CRC pulmonary metastases. Reported mean values of TDT range between 109 (Spratt and Spratt, 1964) and 129 days (Tomimaru et al., 2018). In our case, the growth is faster than this reported range. It is close to the TDT range reported for liver metastases, which are known as more aggressive (Nomura et al., 1998). In summary, the notable aggressiveness of metastases in this case, after two surgeries, supports the assumption that each event of surgery leads to faster of metastases.

*The natural history of the metastases* is evaluated to estimate prognosis and develop an optimal individualized treatment plan. In this case, metastasis formation time (T) and the earliest possible detection time (D) were restored from models of growth in a later period. Note that T can be related to as a parameter of the model, and it can be negative or positive (i.e., before or after primary tumor detection time).

Backward extrapolation of the growth models fitted to data of a later period (after the surgeries) suggests that at least three of the four metastases were seeded about 4 years before, yet could not have been discovered until 1.7 years after primary tumor resection, at the earliest (Figure 2). This extrapolated result is true if the growth rate remains the same from the time of metastasis inception. However, this is quite unlikely, as the implications of the two resections, which were discussed above, may cause acceleration of the metastasis growth. If we consider our model to be correct only for the time *after* the second surgery and assume that growth was *slower* before this surgery, T would be even earlier (although D would not be affected). Therefore, the T values extrapolated here represent the *latest possible* estimated time of metastasis formation.

Formerly published works (Benzekry et al., 2014; Bilous et al., 2019; Vaghi et al., 2020) suggest that the Gompertzian model describes best the metastatic growth, and that considering Gompertzian growth instead of exponential may change extrapolation results, as the curves differ greatly at early times. However, in our case, the Gompertzian models for metastases #1–3 degenerate into exponent, which means that our data is given in an early period of time in the metastatic process when metastasis sizes had not reached their capacity. The Gompertzian model in this period coincides with the exponential curve; therefore, it would make no difference in the predicted value of T (see Supplementary Table 2). Hence, we can conclude that metastases were seeded about 4 years before disease detection and stayed occult until 2 years after it. This result agrees with the empirically supported notion that many metastases are seeded before the primary tumor is even detectable (Hanahan and Weinberg, 2011; Siegel et al., 2017). Lately, analysis of genomic exome-sequencing data has shown that liver and brain metastases in CRC are probably seeded at early stages of disease (Hu et al., 2019).

This work is based on the data of a single patient. Different growth patterns might apply to other cases with different primary tumors and metastatic sites because inter-patient variability is high. Since such clinical data of untreated metastasis growth is not common, robust conclusions on the metastatic growth pattern are difficult to achieve. An example of a way to deal with this challenge is a population model approach, which was used lately by Benzekry et al. to analyze clinical data from brain metastases in NSCLC (Bilous et al., 2019). Their model, comprising of metastasis dissemination and size distribution as a function of primary tumor size, suggests the Gompertzian growth law as most suitable to the data. Like in our case, it is shown that metastases have already been disseminated, but were still occult at the time of disease detection. Unlike in our case, the choice of Gompertzian model makes a significant difference in predicting metastasis onset time, because it differs from exponential curve at early times. This result may suggest that Gompertzian growth is more appropriate to use for prediction of metastasis natural history. Yet, it was achieved for a different type of cancer, and it is still necessary to analyze the clinical data of CRC pulmonary metastases, and more specifically, the data of different subtypes of CRC lung metastases, in order to understand the metastatic process in the relevant type of disease.

In order to reveal how personal characteristics affect metastasis growth pattern, different cases should be studied, thus the unique data of the test case published here are valuable. These data may be used for further analyses with different modeling methods. We intend to do this with a model that includes metastatic dissemination as a function of primary tumor size in a future publication.

In summary, from the unique clinical data of metastasis growth dynamics, we conclude that:

- Untreated lung metastasis growth is logistic, but in most cases, exponential law is a legitimate approximation, as metastases do not verge on the limitation on lesion growth.
- Metastases can be initiated before the primary tumor is detectable (in this case, at least 4–5 years before the primary tumor was detected).
- Surgical removal of the primary tumor or metastasectomy might lead to faster-growing metastases. This is implied by notably short TDT in this case after two surgeries.

## Data Availability Statement

All datasets generated for this study are included in the article/Supplementary Material.

## Ethics Statement

Ethical review and approval was not required for the study on human participants in accordance with the local legislation and institutional requirements. The patients/participants provided their written informed consent to participate in this study. Written informed consent was obtained from the individual(s) for the publication of any potentially identifiable images or data included in this article.

## Author Contributions

SB-M designed the research. GH and SRo performed the research. SB-M and GH contributed analytic tools. SB-M, GH, and ES-S analyzed the data. SRa measured the metastases. GH and ES-S wrote the paper. All authors contributed to the article and approved the submitted version.

## Conflict of Interest

The authors declare that the research was conducted in the absence of any commercial or financial relationships that could be construed as a potential conflict of interest.
