# Supplementary material for: Metastasis Initiation Precedes Detection of Primary Cancer—Analysis of Metastasis Growth in vivo in a Colorectal Cancer Test Case
Source: Front Physiol. 2020 Dec 17;11:533101. doi: 10.3389/fphys.2020.533101 (PMC7773782; doi:10.3389/fphys.2020.533101)
Supplement: Supplementary file 1 [file Data_Sheet_1.docx]

**Supplementary Table S1**

|  | W1 | W2 | W3 | W4 |
| --- | --- | --- | --- | --- |
| date | 19/03/2013 | 29/09/2013 | 30/07/2014 | 09/12/2014 |
| met1 | 0.1368 | 0.4261 | 8.8266 | 14.2470 |
| met2 | 0.0415 | 0.0774 | 0.8869 | 3.2590 |
| MET3 | 0.0256 | - | 0.5069 | 3.5305 |
| MET4 | 0.1839 | 0.5196 | 1.6383 | 1.2004 |

**Table S1.** Metastasis sizes measured by CT scans of the patient, at different times, marked W1 to W4. First row is date of the CT scan, and other rows are metastasis volumes in cm^3^.

**Supplementary Table S2**

|  | T_Exp | T_log | T_Gomp | D_Exp | D_Log | D_Gomp |
| --- | --- | --- | --- | --- | --- | --- |
| met1 | -3.85 | -3.26 | -3.74 | 1.88 | 1.97 | 1.89 |
| met2 | -4.05 | -4.05 | -3.95 | 2.34 | 2.34 | 2.34 |
| MET3 | -3.71 | -3.71 | -3.62 | 2.45 | 2.45 | 2.45 |
| MET4 | -13.84 | -4.21 | 0.22 | 0.25 | 1.68 | 1.85 |

**Table S2.** Estimated times of onset of metastases, defined as time of appearance of the first malignant cell (*T*), and estimated times of metastases' earliest possible detection time, defined as time of metastasis size reaching the threshold enabling detection by CT scan (*D*). *T_k_* and *D_k_* values were extrapolated from each of the fitted models. Times are in years from the day of resection of the primary tumor. Colored lines – chosen best-fitted model (see Table 1).

**Supplementary Table S3**

|  | **Model** | **parameter** | **value fitted to real measure** | **median value** | **10% of sorted values** | **90% of sorted values** | **mean value** | **relative standard error (%)** | **Figure** |
| --- | --- | --- | --- | --- | --- | --- | --- | --- | --- |
| Met | Exponential | $N_{0}^{exp}\left[ {cm}^{3} \right]$ | 6.35E-05 | 6.31E-05 | 1.12E-05 | 2.47E-04 | 1.01E-04 | 3.19 | S2 |
| #1 |  | $\lambda\left[ {years}^{-1} \right]$ | 2.874 | 2.871 | 2.539 | 3.296 | 2.897 | 0.31 | S3 |
|  |  | $T\left[ years \right]$ | -3.847 | -3.851 | -4.890 | -2.827 | -3.858 | 0.63 | S4 |
|  |  | $D\left[ years \right]$ | 1.881 | 1.884 | 1.594 | 2.166 | 1.879 | 0.36 | S5 |
|  | Logistic | $N_{0}^{logistic}\left[ {cm}^{3} \right]$ | 2.86E-05 | 2.58E-05 | 1.25E-06 | 2.03E-04 | 6.83E-05 | 4.18 | S7 |
|  |  | $K^{logistic}\left[ {cm}^{3} \right]$ | 39.234 | 40.238 | 17.885 | 1.20E+07 | 1.20E+07 | 18.34 | S8 |
|  |  | $r\left[ {years}^{-1} \right]$ | 3.151 | 3.193 | 2.589 | 4.073 | 3.267 | 0.55 | S9 |
|  |  | $T\left[ years \right]$ | -3.256 | -3.172 | -4.724 | -1.751 | -3.216 | 1.08 | S10 |
|  |  | $D\left[ years \right]$ | 1.969 | 1.986 | 1.633 | 2.295 | 1.973 | 0.39 | S11 |
|  | Gompertz | $N_{0}^{gomp}\left[ {cm}^{3} \right]$ | 5.91E-05 | 5.86E-05 | 1.00E-05 | 2.60E-04 | 9.98E-05 | 3.38 | S13 |
|  |  | $K^{gomp}\left[ {cm}^{3} \right]$ | 2.84E+296 | 2.19E+299 | 1.74E+296 | 1.8E+303 | 6.0E+302 | 8.16 | S14 |
|  |  | $\beta\left[ {years}^{-1} \right]$ | 4.21E-03 | 4.16E-03 | 3.63E-03 | 4.83E-03 | 4.20E-03 | 0.34 | S15 |
|  |  | $T\left[ years \right]$ | -3.737 | -3.739 | -4.860 | -2.730 | -3.759 | 0.66 | S16 |
|  |  | $D\left[ years \right]$ | 1.885 | 1.888 | 1.570 | 2.167 | 1.881 | 0.37 | S17 |
| Met | Exponential | $N_{0}^{exp}\left[ {cm}^{3} \right]$ | 3.42E-05 | 2.91E-05 | 1.92E-06 | 2.75E-04 | 9.10E-05 | 5.05 | S2 |
| #2 |  | $\lambda\left[ {years}^{-1} \right]$ | 2.578 | 2.618 | 2.057 | 3.278 | 2.651 | 0.54 | S3 |
|  |  | $T\left[ years \right]$ | -4.050 | -3.928 | -6.084 | -2.306 | -9.42E+09 | 49.92 | S4 |
|  |  | $D\left[ years \right]$ | 2.337 | 2.365 | 1.899 | 2.716 | -1.30E+09 | 49.93 | S5 |
|  | Logistic | $N_{0}^{logistic}\left[ {cm}^{3} \right]$ | 3.42E-05 | 3.29E-05 | 8.68E-07 | 2.63E-04 | 9.24E-05 | 5.17 | S7 |
|  |  | $K^{logistic}\left[ {cm}^{3} \right]$ | 7.10E+06 | 1.15E+06 | 9.660 | 7.42E+06 | 7.60E+06 | 13.65 | S8 |
|  |  | $r\left[ {years}^{-1} \right]$ | 2.578 | 2.589 | 2.076 | 3.508 | 2.735 | 0.74 | S9 |
|  |  | $T\left[ years \right]$ | -4.050 | -4.026 | -5.982 | -1.908 | -3.963 | 1.24 | S10 |
|  |  | $D\left[ years \right]$ | 2.337 | 2.354 | 1.912 | 2.725 | 2.339 | 0.42 | S11 |
|  | Gompertz | $N_{0}^{gomp}\left[ {cm}^{3} \right]$ | 3.24E-05 | 2.91E-05 | 1.60E-06 | 2.73E-04 | 1.24E-03 | 47.03 | S13 |
|  |  | $K^{gomp}\left[ {cm}^{3} \right]$ | 6.11E+307 | 2.85E+303 | 1.34E+297 | 6.9E+304 | 2.6E+304 | 8.33 | S14 |
|  |  | $\beta\left[ {years}^{-1} \right]$ | 3.63E-03 | 3.73E-03 | 2.96E-03 | 4.75E-03 | 3.79E-03 | 0.59 | S15 |
|  |  | $T\left[ years \right]$ | -3.951 | -3.868 | -5.911 | -2.188 | -2.62E+07 | 49.93 | S16 |
|  |  | $D\left[ years \right]$ | 2.339 | 2.365 | 1.886 | 2.718 | -4.08E+06 | 49.99 | S17 |
| Met | Exponential | $N_{0}^{exp}\left[ {cm}^{3} \right]$ | 2.03E-05 | 1.76E-05 | 1.94E-07 | 3.13E-04 | 9.62E-05 | 5.45 | S2 |
| #3 |  | $\lambda\left[ {years}^{-1} \right]$ | 2.671 | 2.710 | 2.013 | 3.773 | 2.817 | 0.73 | S3 |
|  |  | $T\left[ years \right]$ | -3.713 | -3.611 | -6.345 | -1.387 | -3.716 | 1.55 | S4 |
|  |  | $D\left[ years \right]$ | 2.451 | 2.484 | 1.869 | 2.964 | 2.452 | 0.51 | S5 |
|  | Logistic | $N_{0}^{logistic}\left[ {cm}^{3} \right]$ | 2.03E-05 | 1.63E-05 | 2.04E-07 | 3.36E-04 | 9.54E-05 | 5.29 | S7 |
|  |  | $K^{logistic}\left[ {cm}^{3} \right]$ | 2.35E+07 | 1.14E+06 | 6.08E+04 | 6.51E+06 | 6.12E+06 | 12.79 | S8 |
|  |  | $r\left[ {years}^{-1} \right]$ | 2.671 | 2.710 | 2.005 | 3.744 | 2.806 | 0.73 | S9 |
|  |  | $T\left[ years \right]$ | -3.714 | -3.594 | -6.376 | -1.428 | -3.733 | 1.53 | S10 |
|  |  | $D\left[ years \right]$ | 2.451 | 2.482 | 1.854 | 2.958 | 2.450 | 0.51 | S11 |
|  | Gompertz | $N_{0}^{gomp}\left[ {cm}^{3} \right]$ | 1.92E-05 | 2.35E-05 | 2.20E-07 | 3.44E-04 | 1.06E-04 | 5.01 | S13 |
|  |  | $K^{gomp}\left[ {cm}^{3} \right]$ | 6.30E+307 | 3.70E+303 | 4.86E+302 | 3.4E+304 | 1.7E+304 | 8.26 | S14 |
|  |  | $\beta\left[ {years}^{-1} \right]$ | 3.76E-03 | 3.75E-03 | 2.83E-03 | 5.33E-03 | 3.91E-03 | 0.74 | S15 |
|  |  | $T\left[ years \right]$ | -3.622 | -3.777 | -6.312 | -1.413 | -3.835 | 1.49 | S16 |
|  |  | $D\left[ years \right]$ | 2.451 | 2.412 | 1.851 | 2.938 | 2.408 | 0.52 | S17 |
| Met | Exponential | $N_{0}^{exp}\left[ {cm}^{3} \right]$ | 1.06E-02 | 9.99E-03 | 2.15E-03 | 4.04E-02 | 1.63E-02 | 3.39 | S2 |
| #4 |  | $\lambda\left[ {years}^{-1} \right]$ | 1.169 | 1.184 | 0.829 | 1.561 | 1.195 | 0.75 | S3 |
|  |  | $T\left[ years \right]$ | -13.839 | -13.589 | -21.014 | -9.407 | -14.539 | 1.05 | S4 |
|  |  | $D\left[ years \right]$ | 0.249 | 0.295 | -1.238 | 1.210 | 0.115 | 27.00 | S5 |
|  | Logistic | $N_{0}^{logistic}\left[ {cm}^{3} \right]$ | 1.29E-04 | 8.90E-05 | 1.81E-07 | 8.24E-03 | 2.60E-03 | 7.70 | S7 |
|  |  | $K^{logistic}\left[ {cm}^{3} \right]$ | 1.523 | 1.563 | 1.136 | 3.481 | 3.36E+06 | 85.77 | S8 |
|  |  | $r\left[ {years}^{-1} \right]$ | 2.795 | 2.916 | 1.311 | 5.050 | 3.074 | 1.42 | S9 |
|  |  | $T\left[ years \right]$ | -4.211 | -3.912 | -11.983 | -1.042 | -5.303 | 2.68 | S10 |
|  |  | $D\left[ years \right]$ | 1.682 | 1.706 | 0.414 | 2.241 | 1.496 | 1.61 | S11 |
|  | Gompertz | $N_{0}^{gomp}\left[ {cm}^{3} \right]$ | 9.23E-12 | 3.48E-28 | 0 | 4.76E-03 | 1.60E-03 | 10.05 | S13 |
|  |  | $K^{gomp}\left[ {cm}^{3} \right]$ | 2.808 | 2.014 | 1.212 | 1.40E+04 | 7.67E+08 | 20.73 | S14 |
|  |  | $\beta\left[ {years}^{-1} \right]$ | 0.869 | 1.250 | 0.107 | 2.757 | 1.402 | 2.28 | S15 |
|  |  | $T\left[ years \right]$ | 0.224 | 0.871 | -6.616 | 1.838 | -0.529 | 21.53 | S16 |
|  |  | $D\left[ years \right]$ | 1.852 | 1.876 | 0.641 | 2.267 | 1.677 | 1.27 | S17 |

**Table S3.** Sensitivity analysis of each of the three models to measured data, for each of the four examined metastases. We have simulated 1000 random samples of artificial data, uniformly distributed within the measurement error bars (calculated according to estimated error of ±2mm in each dimension of the measured lesion, assumed to be spherical). The models were fitted to these data samples, and the distribution of the resulting fitted parameter values was analyzed, as well as the distribution of the estimated times for metastasis formation ($T_{k}$) and metastasis earliest detection time ($D_{k}$), which are directly defined by the fitted parameters. Columns 2, 3 and 4 in this table present median value and interdecile range (values of 10% and of 90% of the sorted values) for each of the fitted parameters. For comparison, the first column presents the results of the same models fitted to the real measured data (same as in Tables 1 and S2). Mean values and relative standard error are also presented (columns 5 and 6), although the distribution is not necessarily symmetric, and for some cases, outliers cause the mean value to be very far from the interdecile range. The last column is referencing to the relevant supplementary figure showing the distribution of parameter values.
