# Supplementary material for: Metastasis Initiation Precedes Detection of Primary Cancer—Analysis of Metastasis Growth in vivo in a Colorectal Cancer Test Case
Source: Front Physiol. 2020 Dec 17;11:533101. doi: 10.3389/fphys.2020.533101 (PMC7773782; doi:10.3389/fphys.2020.533101)
Supplement: Supplementary file 2 [file Data_Sheet_2.docx]

***Sensitivity Analysis Figures***


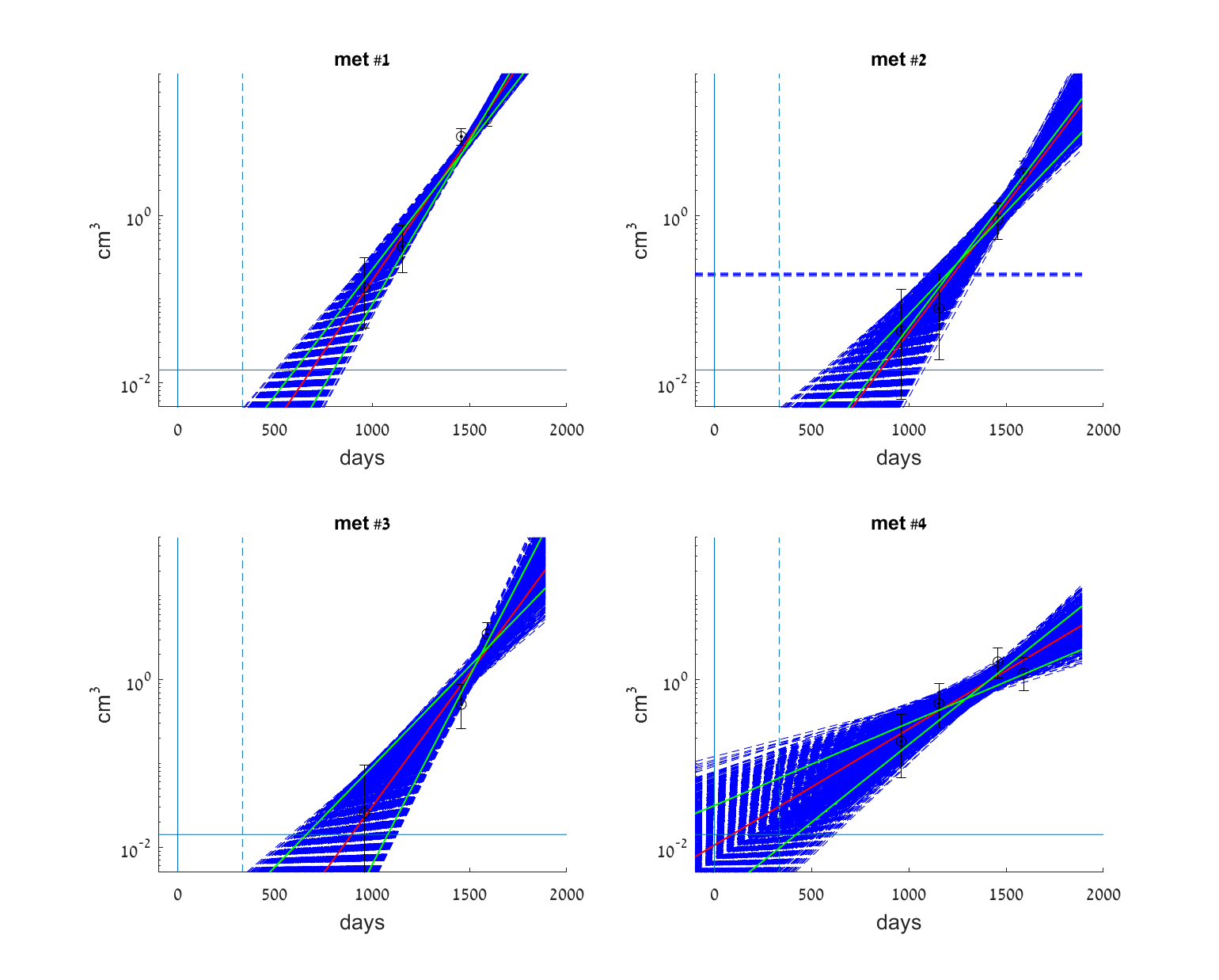


***Figure S1: Exponential model - Sensitivity analysis of the fit. Blue dashed lines: exponential models, fitted to a 1000 random different measurement samples within the measured data error bars. Black circles: Clinical data measurements with measurement error bars. Red curve: The model fitted to the reported measurement data. Green curves: interdecile range (fitted models with 10% and 90% of the sorted*** $\lambda$ ***values).***

***
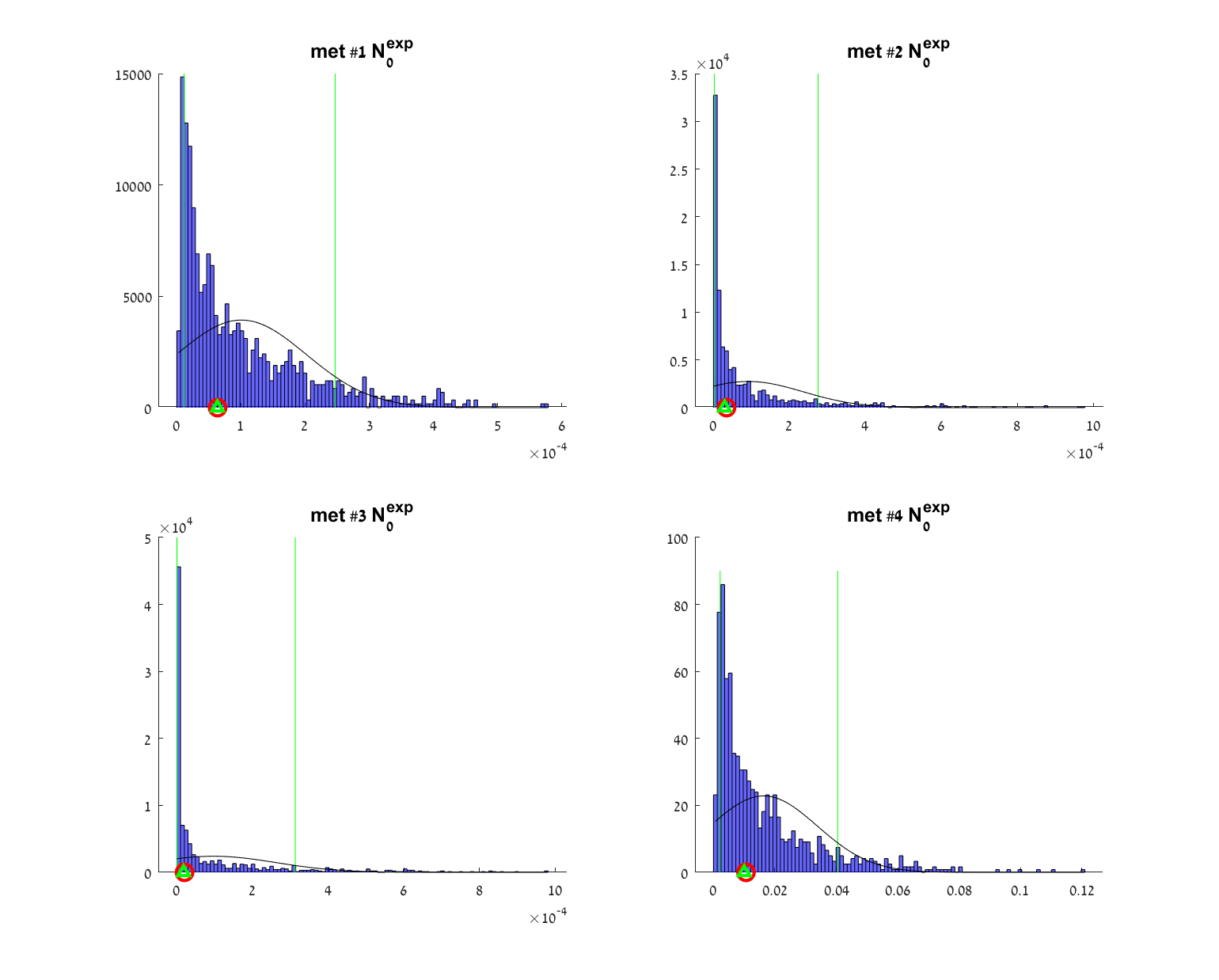
***

***Figure S2: Exponential model - Sensitivity analysis of the fit. Blue bars: A histogram of*** $N_{0}^{exp}$ ***parameter values, fitted to a 1000 random measurement samples within the measured data error bars. Black curve: A PDF plot assuming normal distribution of the*** $N_{0}^{exp}$ ***values. Green triangle: Median*** $N_{0}^{exp}$ ***value. Green vertical lines: interdecile range (10% and 90% of the sorted*** $N_{0}^{exp}$ ***values). Red circle: The*** $N_{0}^{exp}$ ***value fitted to the reported measured data.***

***
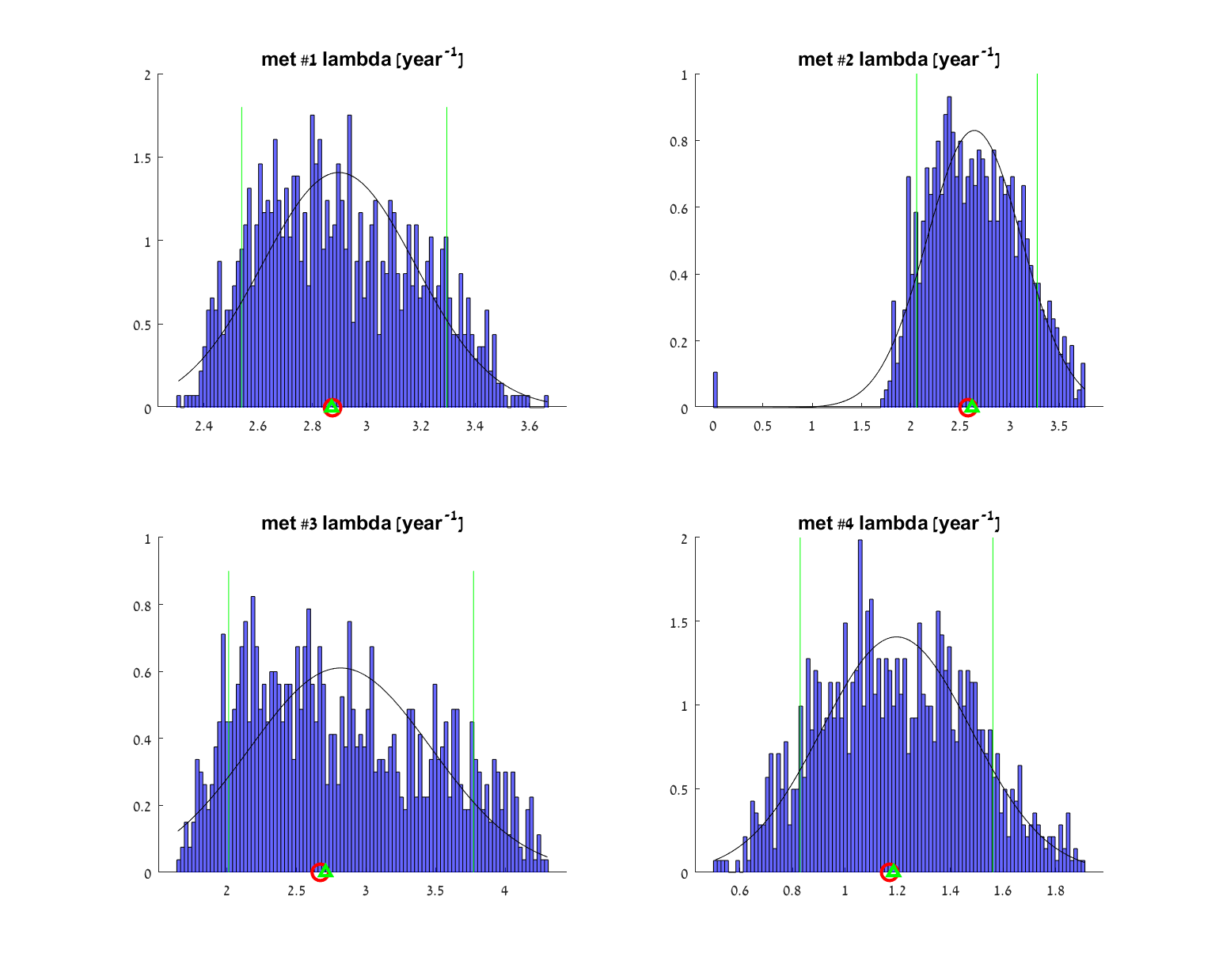
***

***Figure S3: Exponential model - Sensitivity analysis of the fit. Blue bars: A histogram of*** $\lambda$ ***parameter values, fitted to a 1000 random measurement samples within the measured data error bars. Black curve: A PDF plot assuming normal distribution of the*** $\lambda$ ***values. Green triangle: Median*** $\lambda$ ***value. Green vertical lines: interdecile range (10% and 90% of the sorted*** $\lambda$ ***values). Red circle: The*** $\lambda$ ***value fitted to the reported measured data.***

***
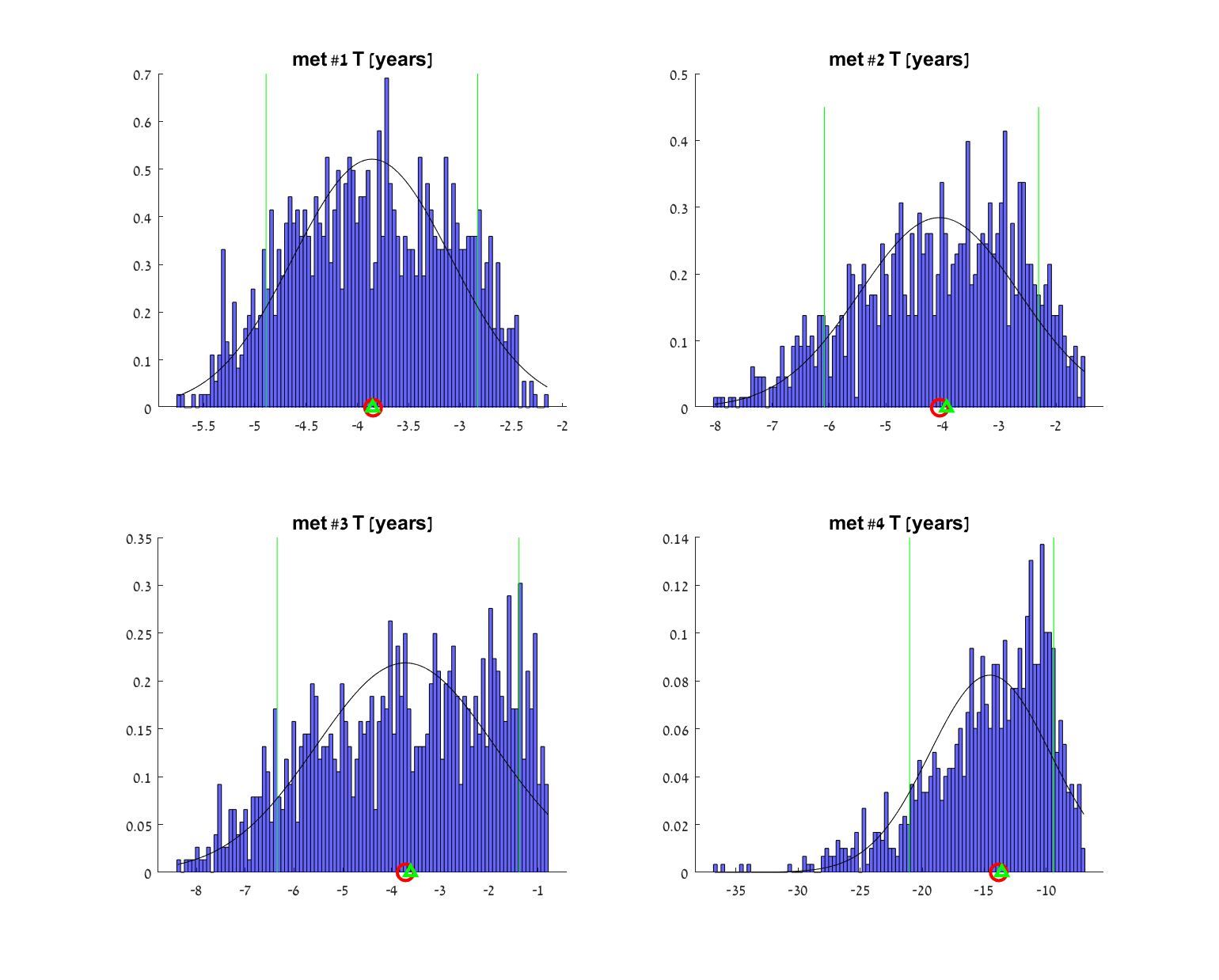
***

***Figure S4: Exponential model - Sensitivity analysis of the fit. Blue bars: A histogram of metastasis onset time (***$T$***) values, calculated from values of model parameters that were fitted to a 1000 random measurement samples within the measured data error bars. Black curve: A PDF plot assuming normal distribution of the*** $T$ ***values. Green triangle: Median*** $T$ ***value. Green vertical lines: interdecile range (10% and 90% of the sorted values). Red circle: The*** $T$ ***value resulting from fit to the reported measured data.***

***
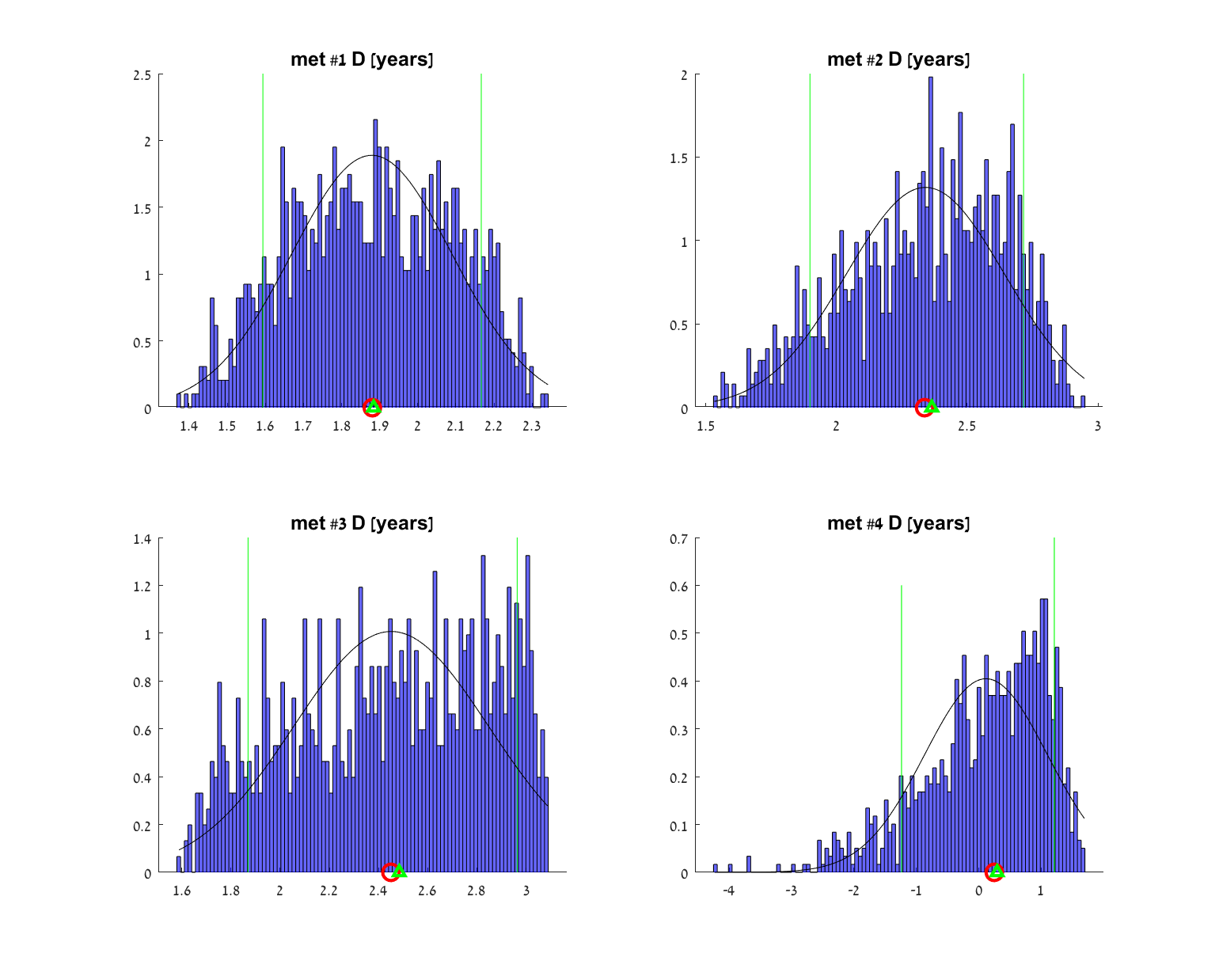
***

***Figure S5: Exponential model - Sensitivity analysis of the fit. Blue bars: A histogram of earliest possible detection time (***$D$***) values, calculated from values of model parameters that were fitted to a 1000 random measurement samples within the measured data error bars. Black curve: A PDF plot assuming normal distribution of the*** $D$ ***values. Green triangle: Median*** $D$ ***value. Green vertical lines: interdecile range (10% and 90% of the sorted values). Red circle: The*** $D$ ***value resulting from fit to the reported measured data.***

***
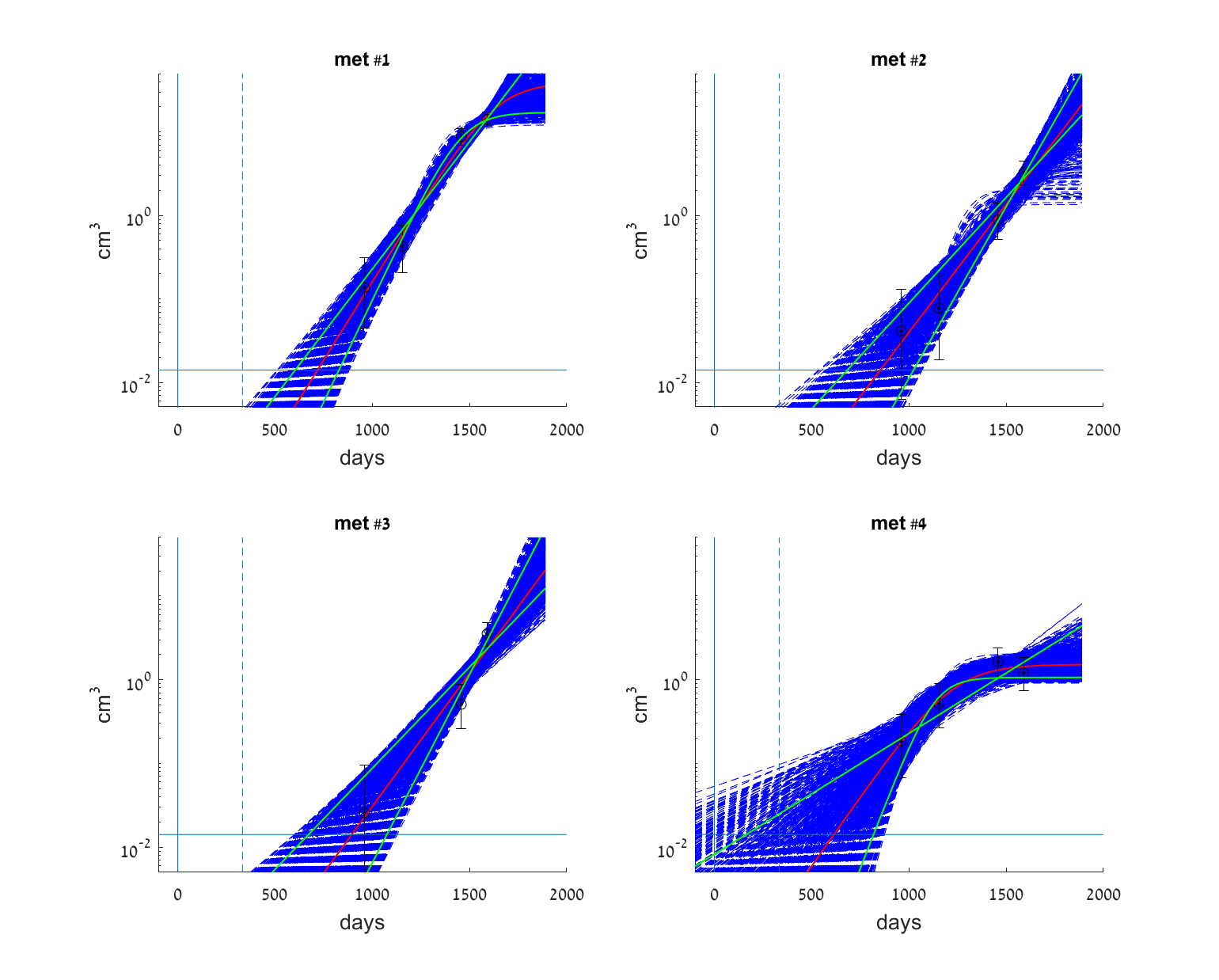
***

***Figure S6: Logistic model - Sensitivity analysis of the fit. Blue dashed lines: logistic models, fitted to a 1000 random different measurement samples within the measured data error bars. Black circles: Clinical data measurements with measurement error bars. Red curve: The model fitted to the reported measurement data. Green curves: interdecile range (fitted models with 10% and 90% of the sorted*** $\lambda$ ***values).***


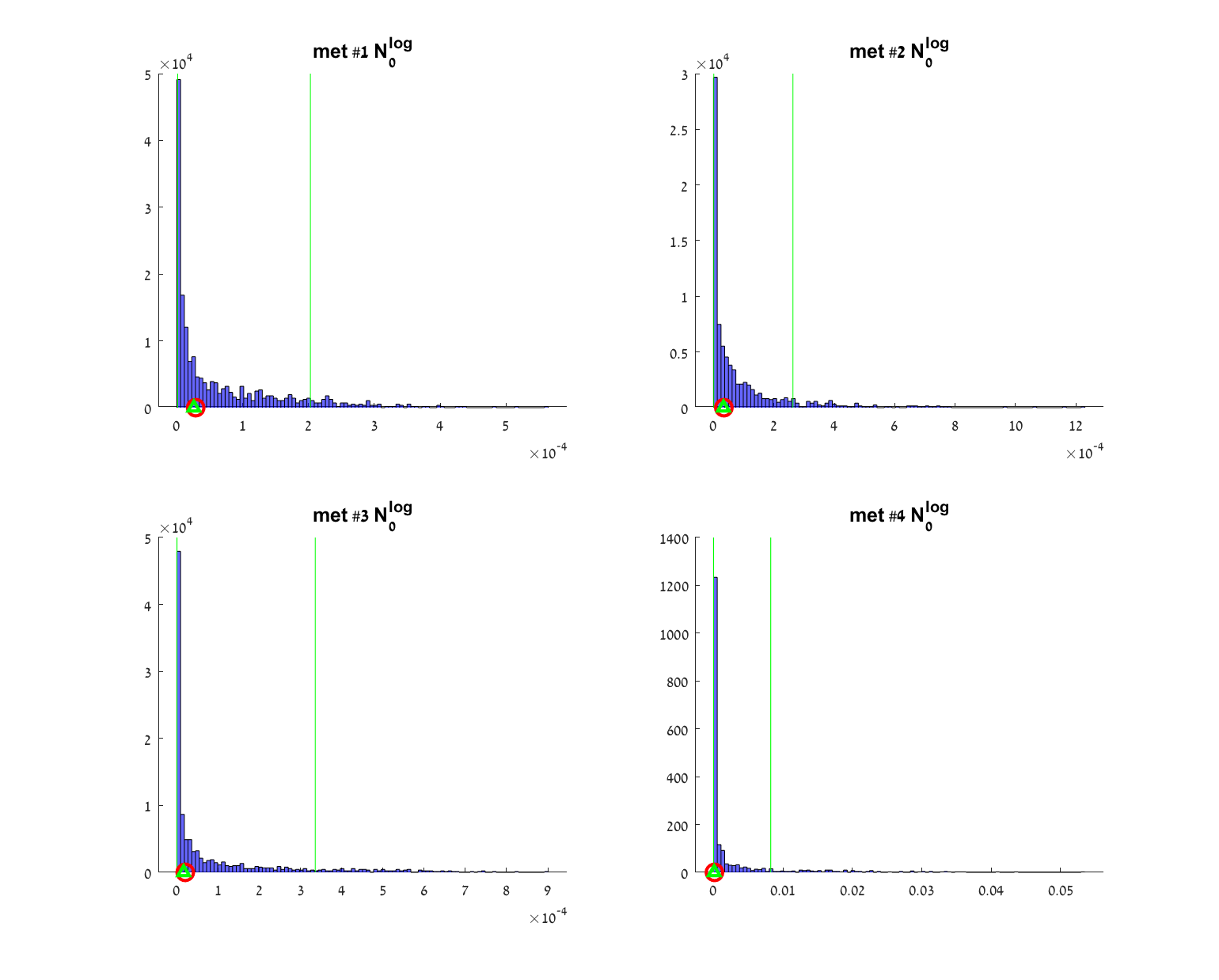


***Figure S7: Logistic model - Sensitivity analysis of the fit. Blue bars: A histogram of*** $N_{0}^{log}$ ***parameter values, fitted to a 1000 random measurement samples within the measured data error bars. Green triangle: Median*** $N_{0}^{log}$ ***value. Green vertical lines: interdecile range (10% and 90% of the sorted*** $N_{0}^{log}$ ***values). Red circle: The*** $N_{0}^{log}$ ***value fitted to the reported measured data.***

***
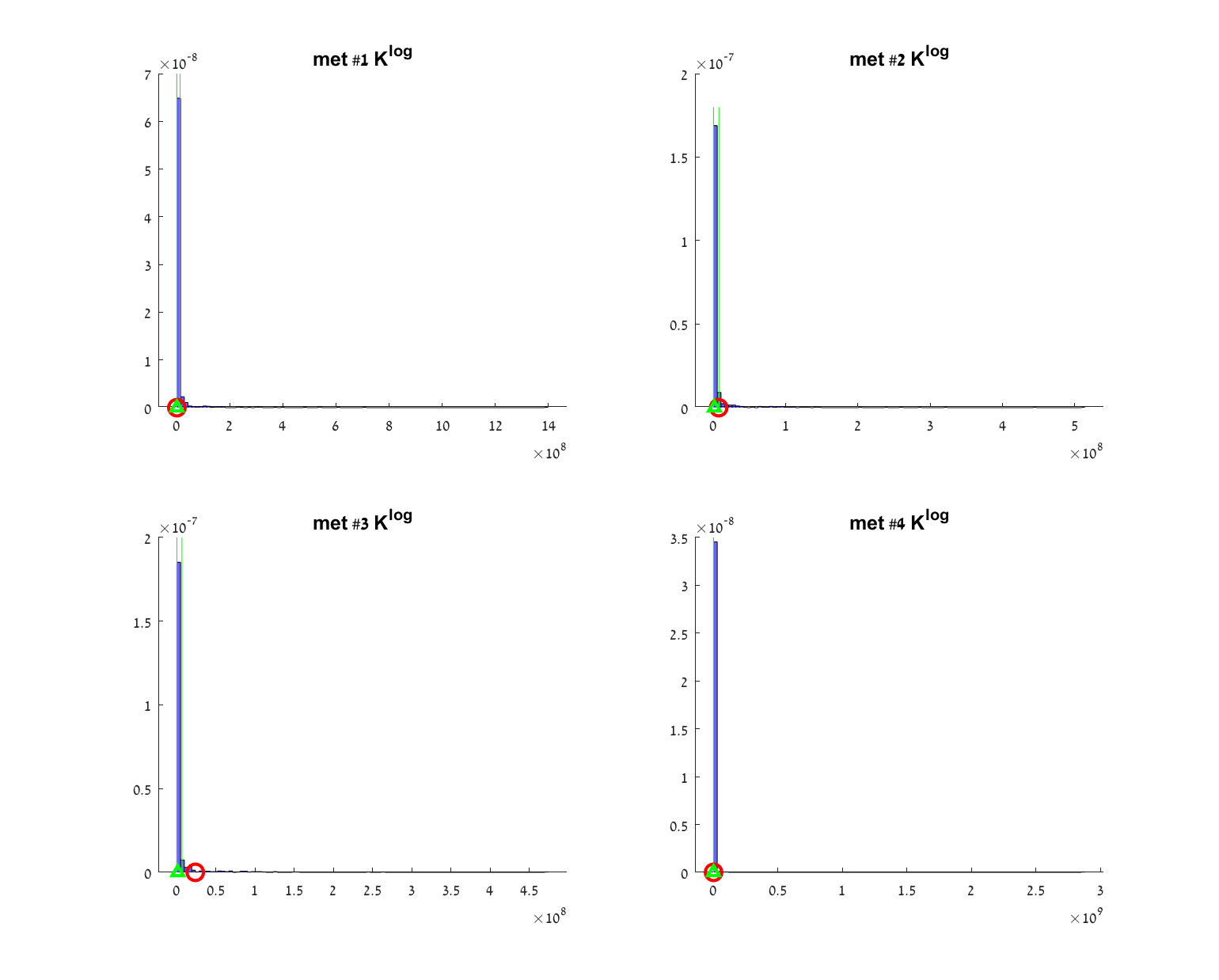
***

***Figure S8: Logistic model - Sensitivity analysis of the fit. Blue bars: A histogram of*** $K^{log}$ ***parameter values, fitted to a 1000 random measurement samples within the measured data error bars. Green triangle: Median*** $K^{log}$ ***value. Green vertical lines: interdecile range (10% and 90% of the sorted*** $K^{log}$ ***values). Red circle: The*** $K^{log}$ ***value fitted to the reported measured data.***

***
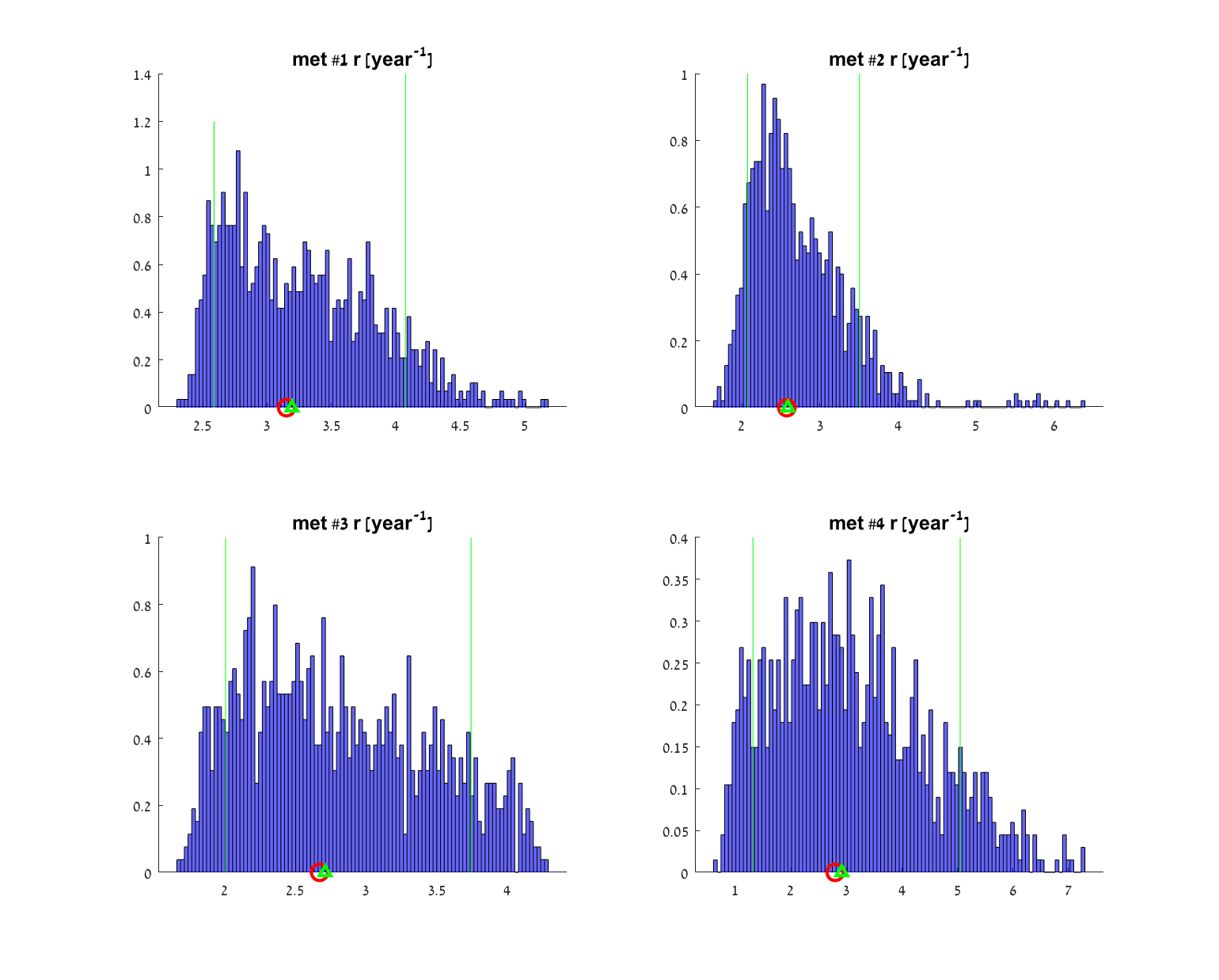
***

***Figure S9: Logistic model - Sensitivity analysis of the fit. Blue bars: A histogram of*** $r$ ***parameter values, fitted to a 1000 random measurement samples within the measured data error bars. Green triangle: Median*** $r$ ***value. Green vertical lines: interdecile range (10% and 90% of the sorted*** $r$ ***values). Red circle: The*** $r$ ***value fitted to the reported measured data.***

***
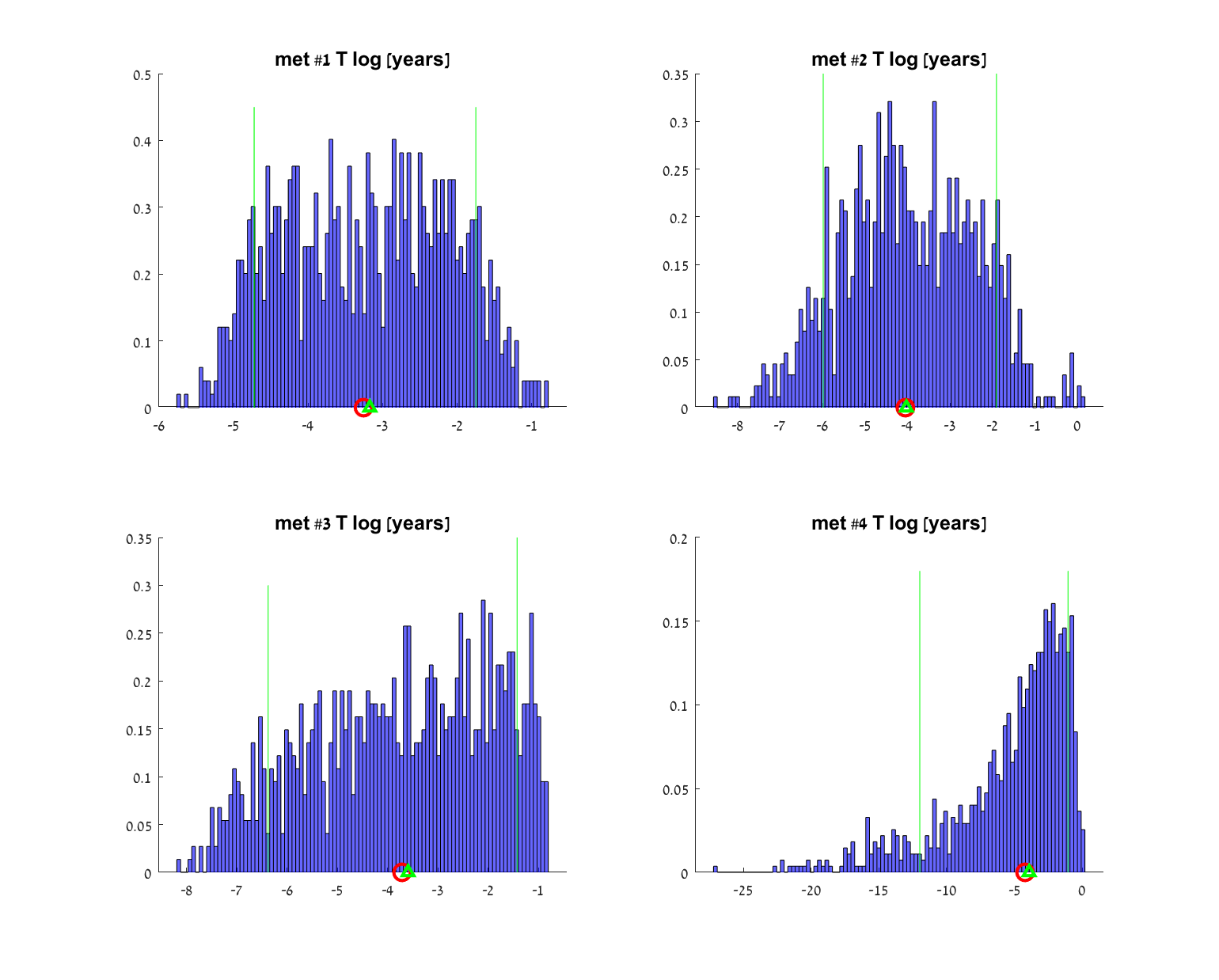
***

***Figure S10: Logistic model - Sensitivity analysis of the fit. Blue bars: A histogram of metastasis onset time (***$T$***) values, calculated from values of model parameters that were fitted to a 1000 random measurement samples within the measured data error bars. Green triangle: Median*** $T$ ***value. Green vertical lines: interdecile range (10% and 90% of the sorted values). Red circle: The*** $T$ ***value resulting from fit to the reported measured data.***

***
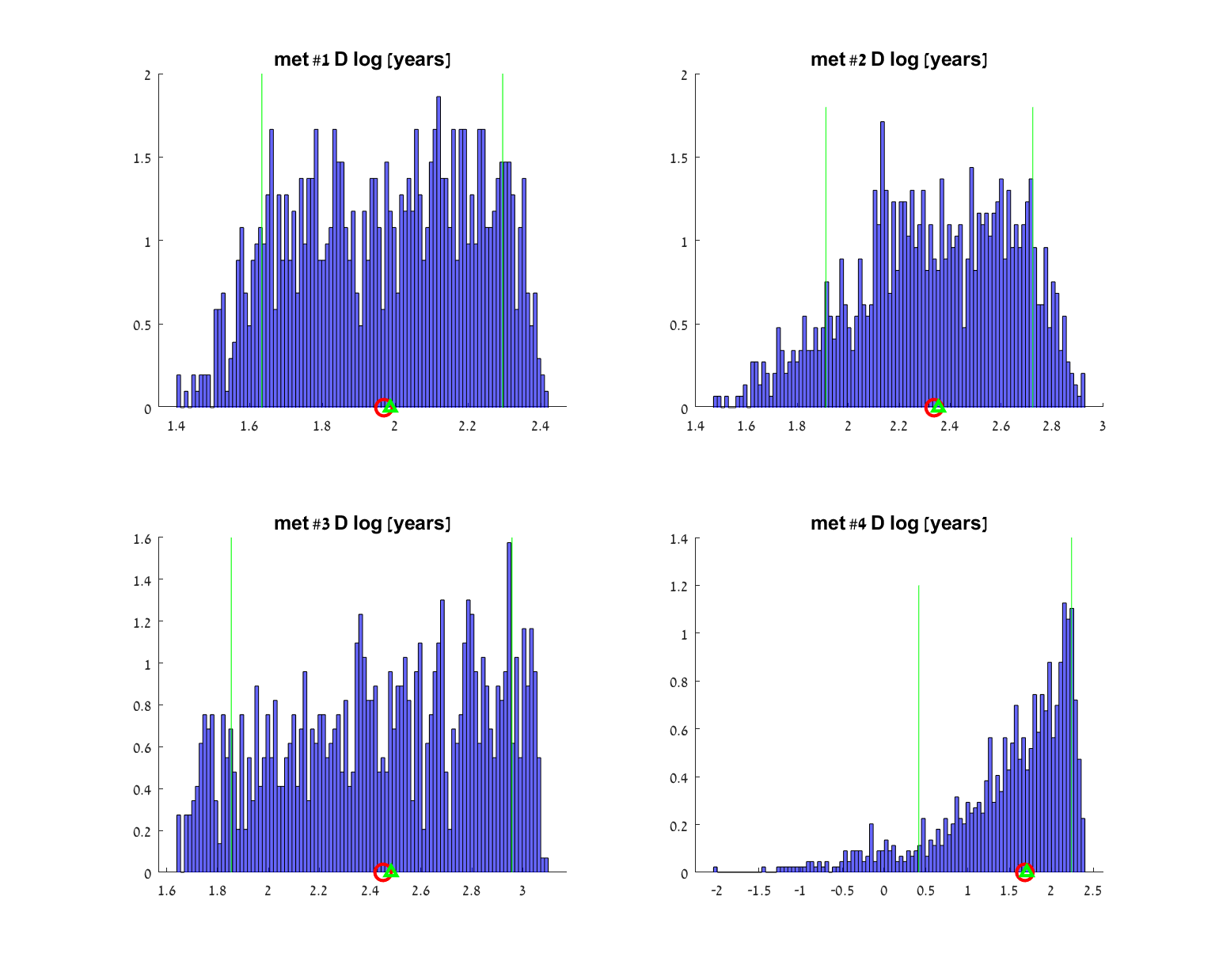
***

***Figure S11: Logistic model - Sensitivity analysis of the fit. Blue bars: A histogram of earliest possible detection time (***$D$***) values, calculated from values of model parameters that were fitted to a 1000 random measurement samples within the measured data error bars. Green triangle: Median*** $D$ ***value. Green vertical lines: interdecile range (10% and 90% of the sorted values). Red circle: The*** $D$ ***value resulting from fit to the reported measured data.***


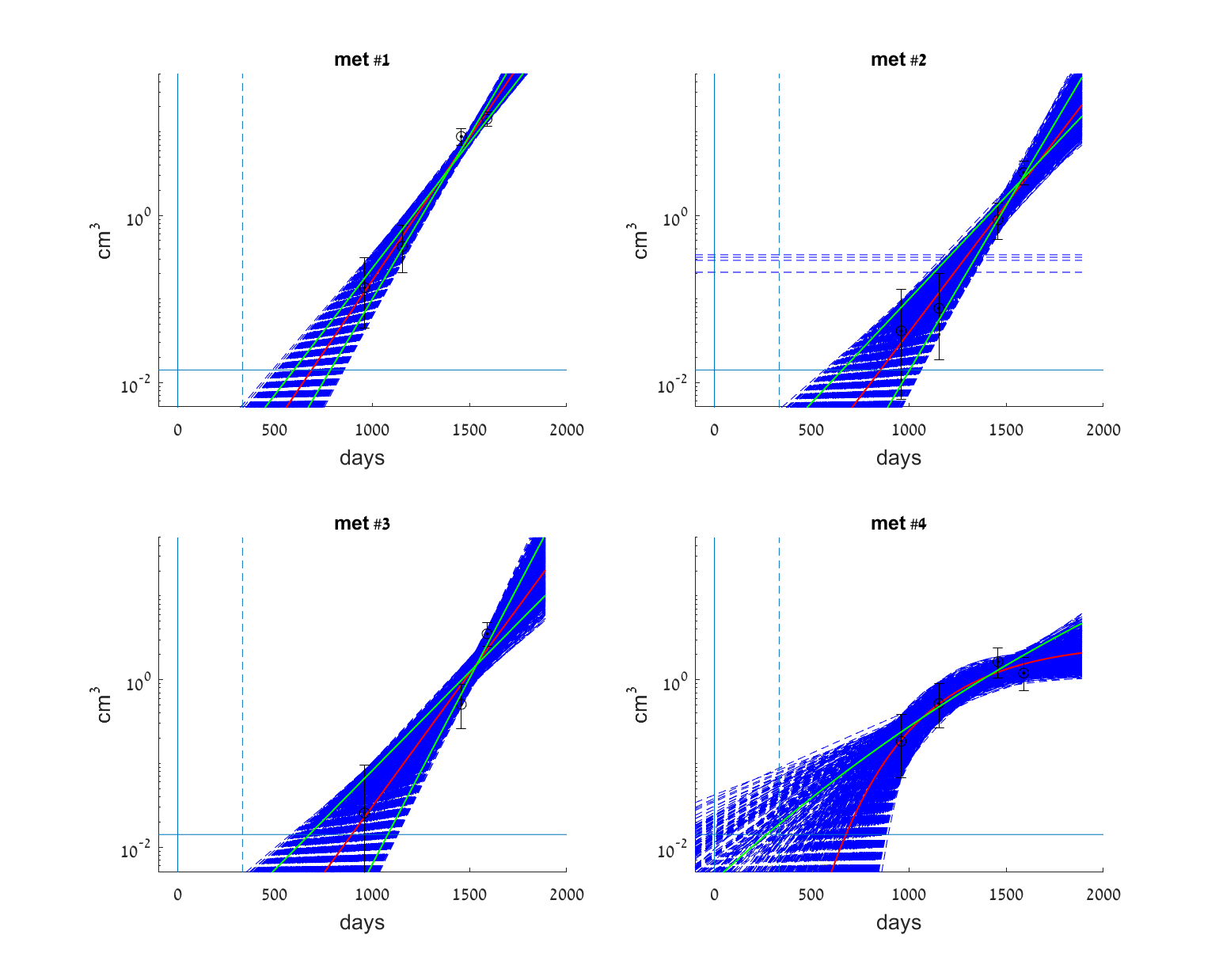


***Figure S12: Gompertz model – Sensitivity analysis of the fit. Blue dashed lines: Gompertz models, fitted to a 1000 random different measurement samples within the measured data error bars. Black circles: Clinical data measurements with measurement error bars. Red curve: The model fitted to the reported measurement data. Green curves: interdecile range (fitted models with 10% and 90% of the sorted*** $\lambda$ ***values).***


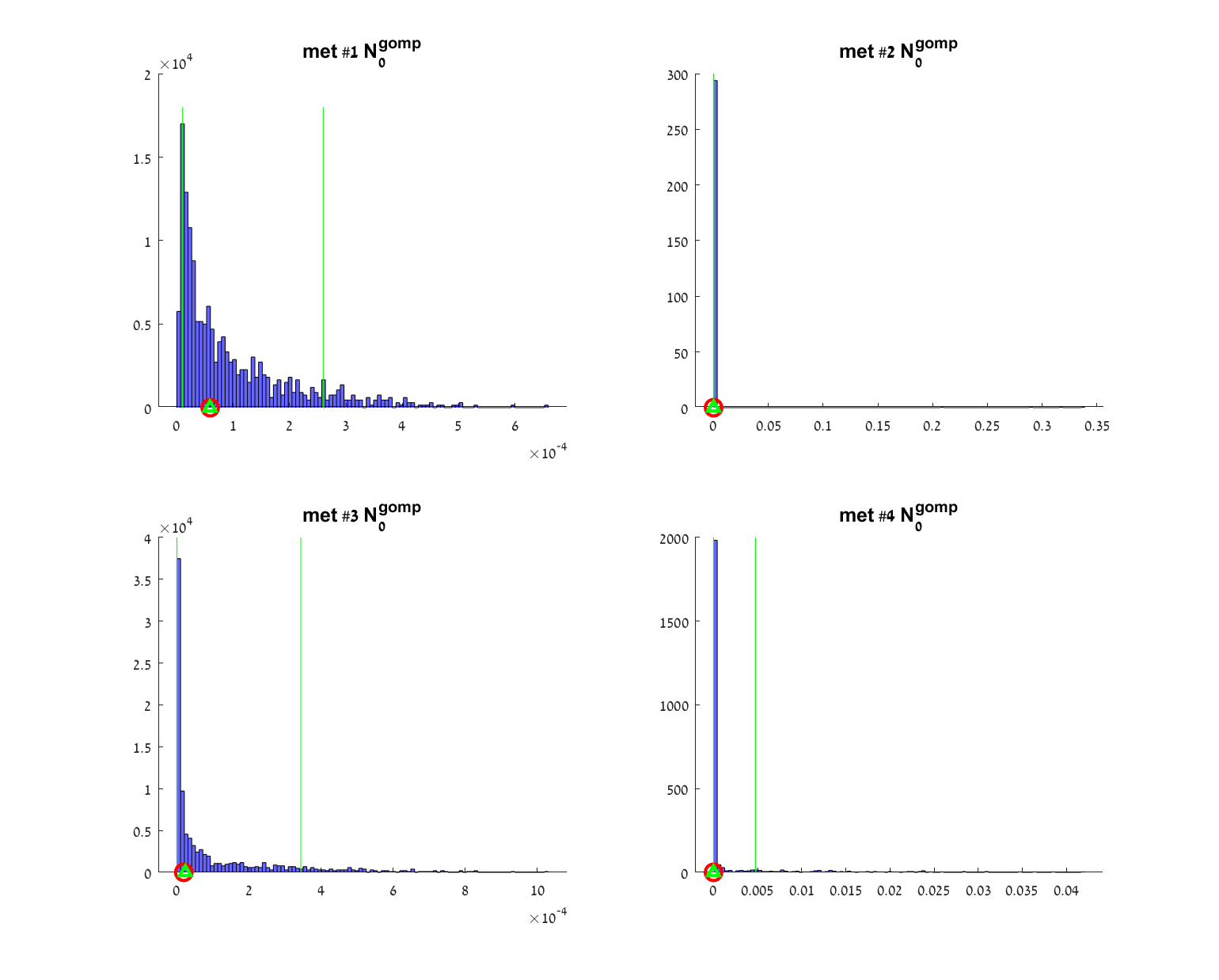


***Figure S13: Gompertz model - Sensitivity analysis of the fit. Blue bars: A histogram of*** $N_{0}^{gomp}$ ***parameter values, fitted to a 1000 random measurement samples within the measured data error bars. Green triangle: Median*** $N_{0}^{gomp}$ ***value. Green vertical lines: interdecile range (10% and 90% of the sorted*** $N_{0}^{gomp}$ ***values). Red circle: The*** $N_{0}^{gomp}$ ***value fitted to the reported measured data.***


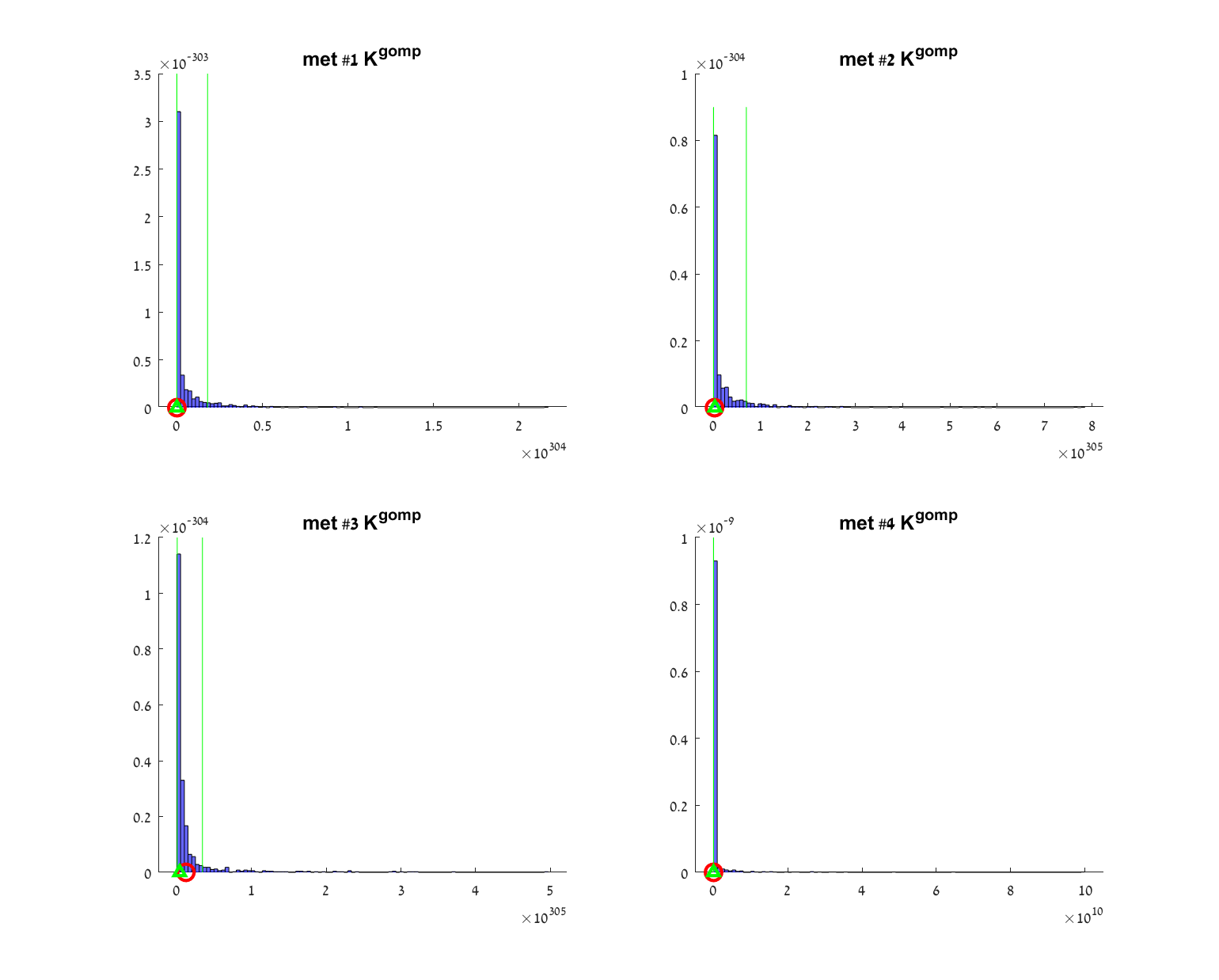


***Figure S14: Gompertz model - Sensitivity analysis of the fit. Blue bars: A histogram of*** $K^{gomp}$ ***parameter values, fitted to a 1000 random measurement samples within the measured data error bars. Green triangle: Median*** $K^{gomp}$ ***value. Green vertical lines: interdecile range (10% and 90% of the sorted*** $K^{gomp}$ ***values). Red circle: The*** $K^{gomp}$ ***value fitted to the reported measured data.***


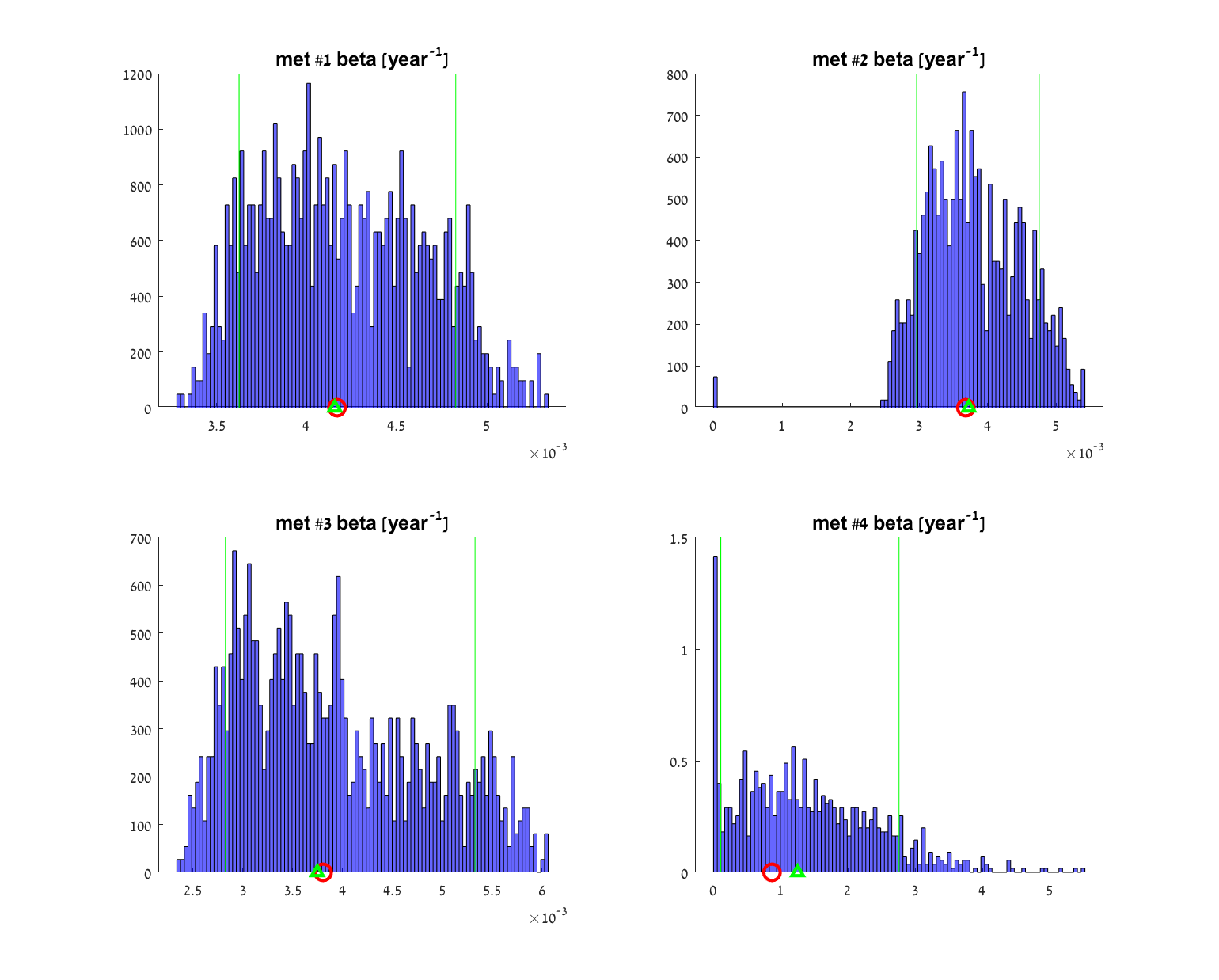


***Figure S15: Gompertz model - Sensitivity analysis of the fit. Blue bars: A histogram of*** $\beta$ ***parameter values, fitted to a 1000 random measurement samples within the measured data error bars. Green triangle: Median*** $\beta$ ***value. Green vertical lines: interdecile range (10% and 90% of the sorted*** $\beta$ ***values). Red circle: The*** $\beta$ ***value fitted to the reported measured data.***


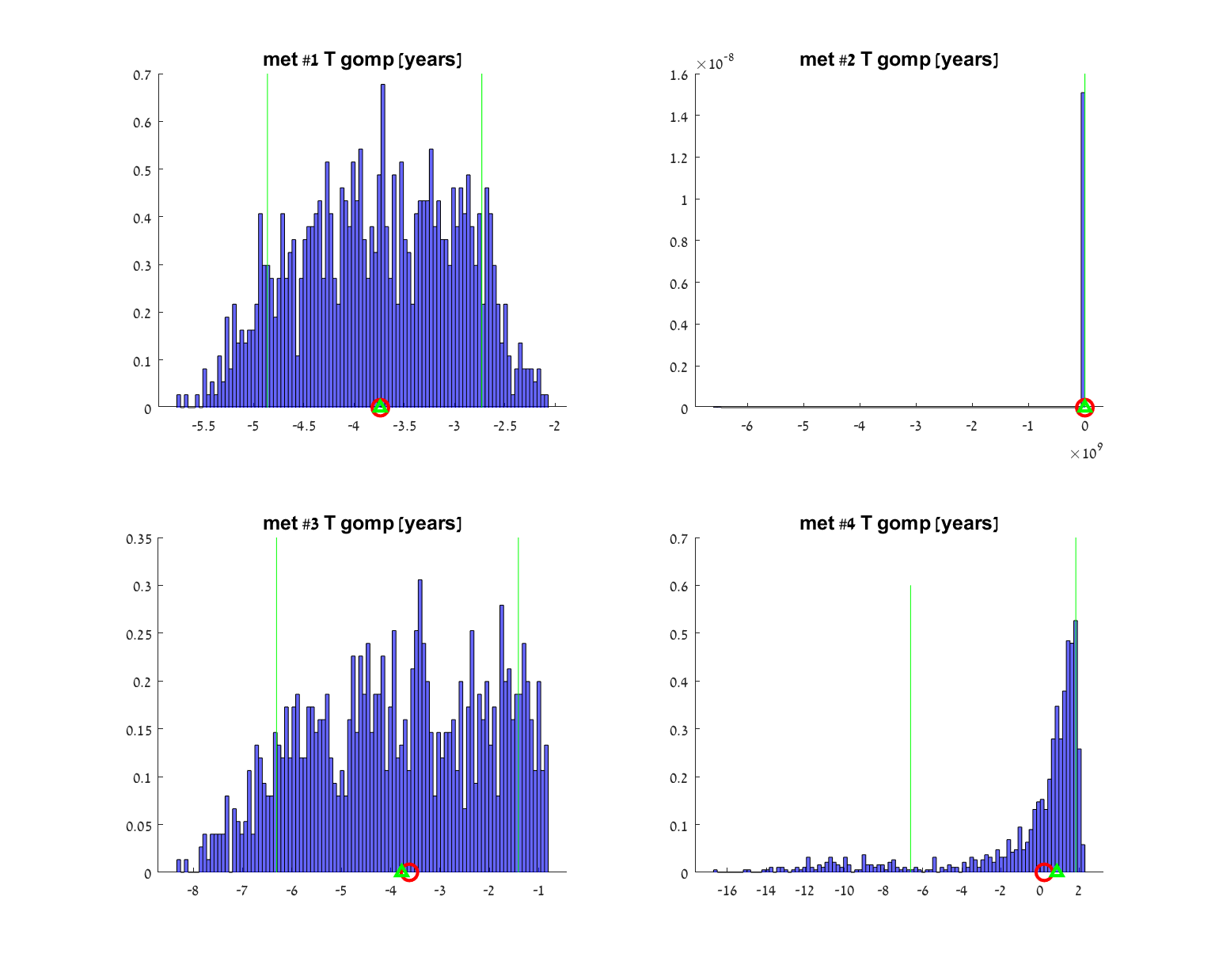


***Figure S16: Gompertz model - Sensitivity analysis of the fit. Blue bars: A histogram of metastasis onset time (***$T$***) values, calculated from values of model parameters that were fitted to a 1000 random measurement samples within the measured data error bars. Green triangle: Median*** $T$ ***value. Green vertical lines: interdecile range (10% and 90% of the sorted values). Red circle: The*** $T$ ***value resulting from fit to the reported measured data.***


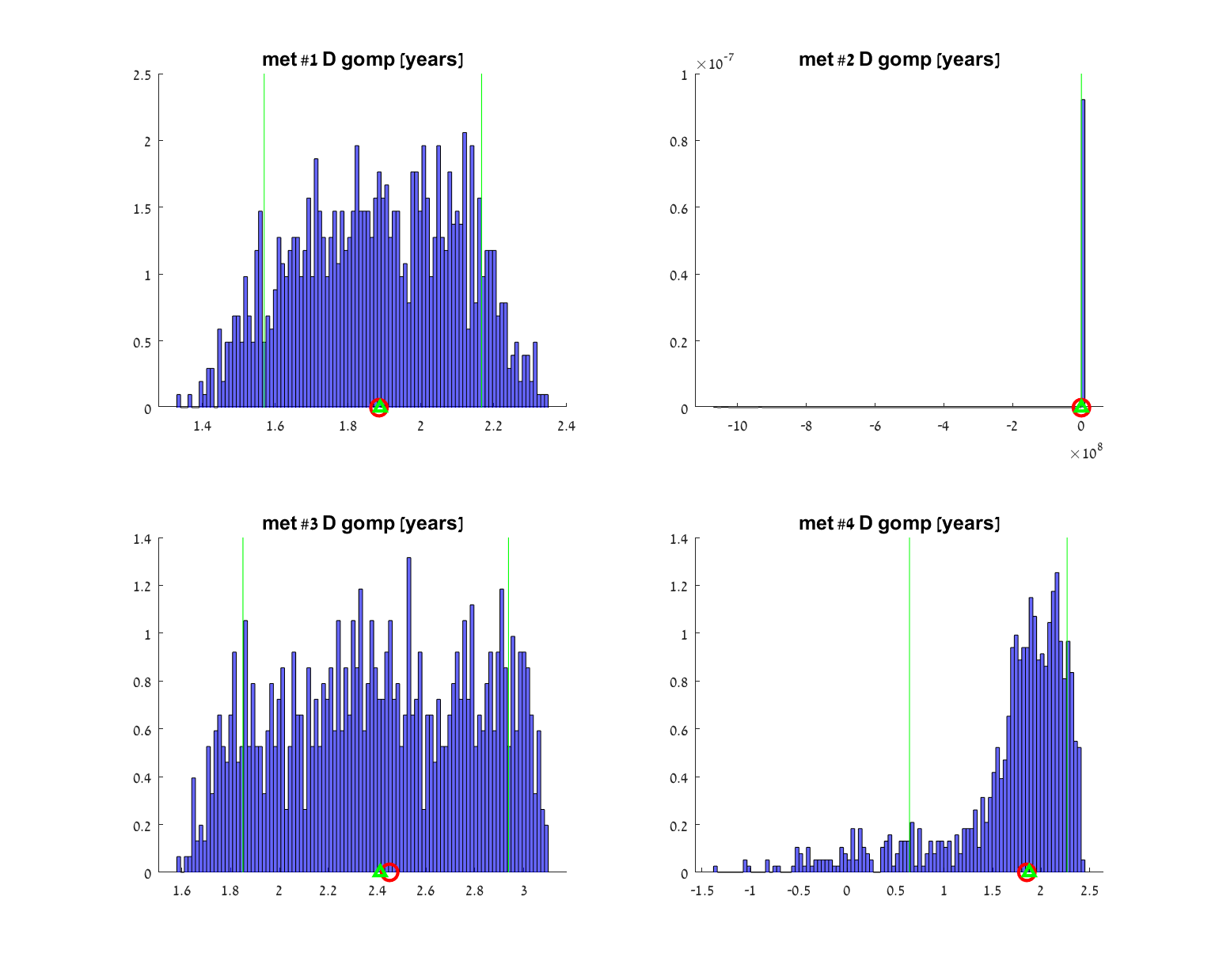


***Figure S17: Gompertz model - Sensitivity analysis of the fit. Blue bars: A histogram of earliest possible detection time (***$D$***) values, calculated from values of model parameters that were fitted to a 1000 random measurement samples within the measured data error bars. Green triangle: Median*** $D$ ***value. Green vertical lines: interdecile range (10% and 90% of the sorted values). Red circle: The*** $D$ ***value resulting from fit to the reported measured data.***
